# Structure-Based Discovery of Mouse Trace Amine-Associated Receptor 5 Antagonists

**DOI:** 10.1021/acs.jcim.3c00755

**Published:** 2023-10-17

**Authors:** Alessandro Nicoli, Verena Weber, Carlotta Bon, Alexandra Steuer, Stefano Gustincich, Raul R. Gainetdinov, Roman Lang, Stefano Espinoza, Antonella Di Pizio

**Affiliations:** † Leibniz Institute for Food Systems Biology at the Technical University of Munich, 85354 Freising, Germany; ‡ Chemoinformatics and Protein Modelling, Department of Molecular Life Sciences, School of Life Sciences, Technical University of Munich, 85354 Freising, Germany; § Institute for Advanced Simulations (IAS)-5/Institute for Neuroscience and Medicine (INM)-9, Forschungszentrum Jülich, 52428 Jülich, Germany; ∥ Faculty of Mathematics, Computer Science and Natural Sciences, RWTH Aachen, Aachen, 52062 Germany; ⊥ Istituto Italiano di Tecnologia, 16163 Genova, Italy; # Institute of Translational Biomedicine and Saint Petersburg University Hospital, Saint Petersburg State University, Saint Petersburg 199034, Russia; ¶ Dipartimento di Scienze della Salute, Università del Piemonte Orientale, 28100 Novara, Italy

## Abstract

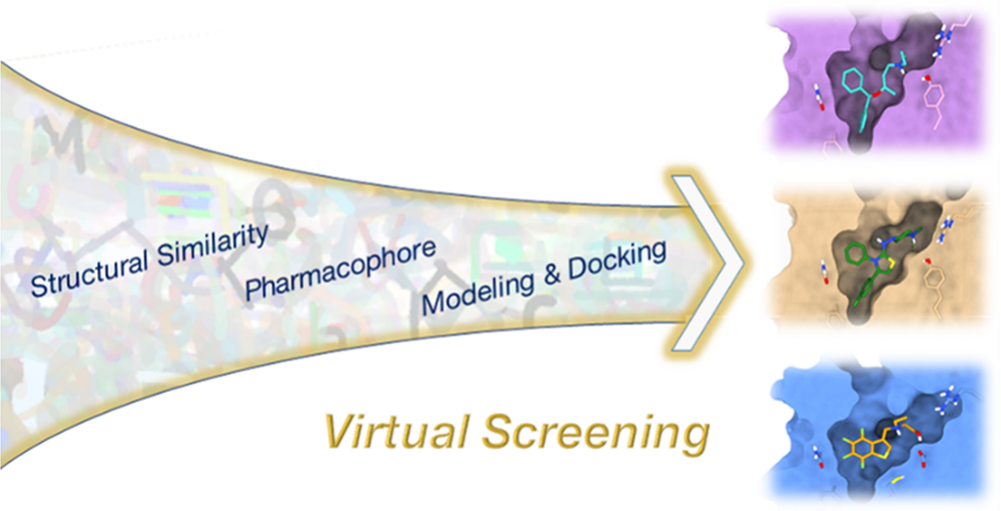

Trace amine-associated receptors (TAARs) were discovered in 2001 as new members of class A G protein-coupled receptors (GPCRs). With the only exception of TAAR1, TAAR members (TAAR2–9, also known as noncanonical olfactory receptors) were originally described exclusively in the olfactory epithelium and believed to mediate the innate perception of volatile amines. However, most noncanonical olfactory receptors are still orphan receptors. Given its recently discovered nonolfactory expression and therapeutic potential, TAAR5 has been the focus of deorphanization campaigns that led to the discovery of a few druglike antagonists. Here, we report four novel TAAR5 antagonists identified through high-throughput screening, which, along with the four ligands published in the literature, constituted our starting point to design a computational strategy for the identification of TAAR5 ligands. We developed a structure-based virtual screening protocol that allowed us to identify three new TAAR5 antagonists with a hit rate of 10%. Despite lacking an experimental structure, we accurately modeled the TAAR5 binding site by integrating comparative sequence- and structure-based analyses of serotonin receptors with homology modeling and side-chain optimization. In summary, we have identified seven new TAAR5 antagonists that could serve as lead candidates for the development of new treatments for depression, anxiety, and neurodegenerative diseases.

## Introduction

Trace amine-associated receptors (TAARs) belong to class A G protein-coupled receptors (GPCRs).^1,2^ Twenty-six subtypes of TAARs have been identified in mammalian species and categorized into nine different subfamilies (TAAR1–9).^3^ The number of TAARs differs by species: humans express only 6 receptor subtypes, mice express 15, and zebrafish express even as many as 112 subtypes.^3,4^ The first deorphanized receptor, TAAR1, was found to respond to the biogenic trace amines (TAs); thus, the receptor subfamily was named after it. The term trace amines was originally defined as endogenous amines with a physiological tissue level below 100 ng/g but is mostly used to describe *p*-tyramine, beta phenylethylamine, tyramine, and *p*-octopamine.^5,6^ Besides, a broad spectrum of biogenic and synthetic amines derivatives are now known as modulators of TAAR1.^6,7^

TAAR2–9 members were initially detected in murine olfactory sensory neurons and therefore defined as noncanonical olfactory receptors. They play a critical role in detecting volatile amines associated with distinct ethological or ecological cues, and their ligands are present in decaying foods and animal body fluids as a consequence of the decarboxylation of amino acids by endogenous enzymes or through microbial metabolism.^8^ TAs mediate innate animal social communication, such as sexual attraction, predator avoidance and aversive response that are crucial for animal survival and reproduction.^9,10^ Recent evidence of the presence of TAAR2–9 members in several extranasal tissues suggests their involvement in physiological processes other than olfaction.^11−14^ TAAR2–9 receptors are now suggested as potential drug targets for several diseases, including food-induced inflammatory responses (e.g., Crohn′s disease, ulcerative colitis), metabolic disorders (e.g., type-2 diabetes, obesity) and even melanoma.^15−20^ However, a detailed characterization of TAAR2–9 is hampered by their low expression levels and the limited number of experimentally identified ligands.^6^

Murine subtypes are the best characterized among mammals in terms of the number of ligands.^6,21−24^ For the mouse TAAR5 (mTAAR5), both agonists (that is trimethylamine, a sexually dimorphic mouse odor secreted into urine,^21,25,26^ and alpha-NETA,^27^ an acetylcholine esterase inhibitor) and antagonists^28^ (two 5-HT_1A_ ligands) are known. Notably, recent studies found that mTAAR5 is expressed in the major limbic brain areas and is involved in the regulation of emotional behavior, suggesting that TAAR5 antagonism may represent a novel therapeutic strategy for anxiety and depression.^13,17,29,30^ Moreover, a correlation between mTAAR5 with adult neurogenesis and dopamine transmission, and its involvement in sensorimotor functions and cognitive processes have been suggested.^17,29−31^ The limited knowledge of the druglike ligand space of TAAR5 modulators impairs potential TAAR5-targeted drug discovery campaigns. Thus, the discovery of additional ligands is a crucial step in gaining a mechanistic and physiological understanding of TAAR5. Interestingly, mTAAR5 and hTAAR5 (human TAAR5) share 87% of sequence identity, and the knowledge gained for mTAAR5 and its ligands could be then transferred to hTAAR5. Structure-based virtual screening campaigns have been successfully applied for GPCR ligand discovery and have proved to be successful also for the deorphanization of mTAAR5.^28,32,33^ Here, we report a structure-based virtual screening protocol that allowed us to identify new mTAAR5 antagonists with a hit rate of 10%.

## Results

In the current work, we present a structure-based virtual screening protocol developed to identify new ligands for the mTAAR5. We combined structure- and sequence-based analyses to characterize the orthosteric binding site of mTAAR5 and then used this information to generate a structural model for a virtual screening campaign. Selected molecules were then experimentally validated in an in vitro BRET assay.^34^

### mTAAR5 Ligand Set

**Figure 1:**
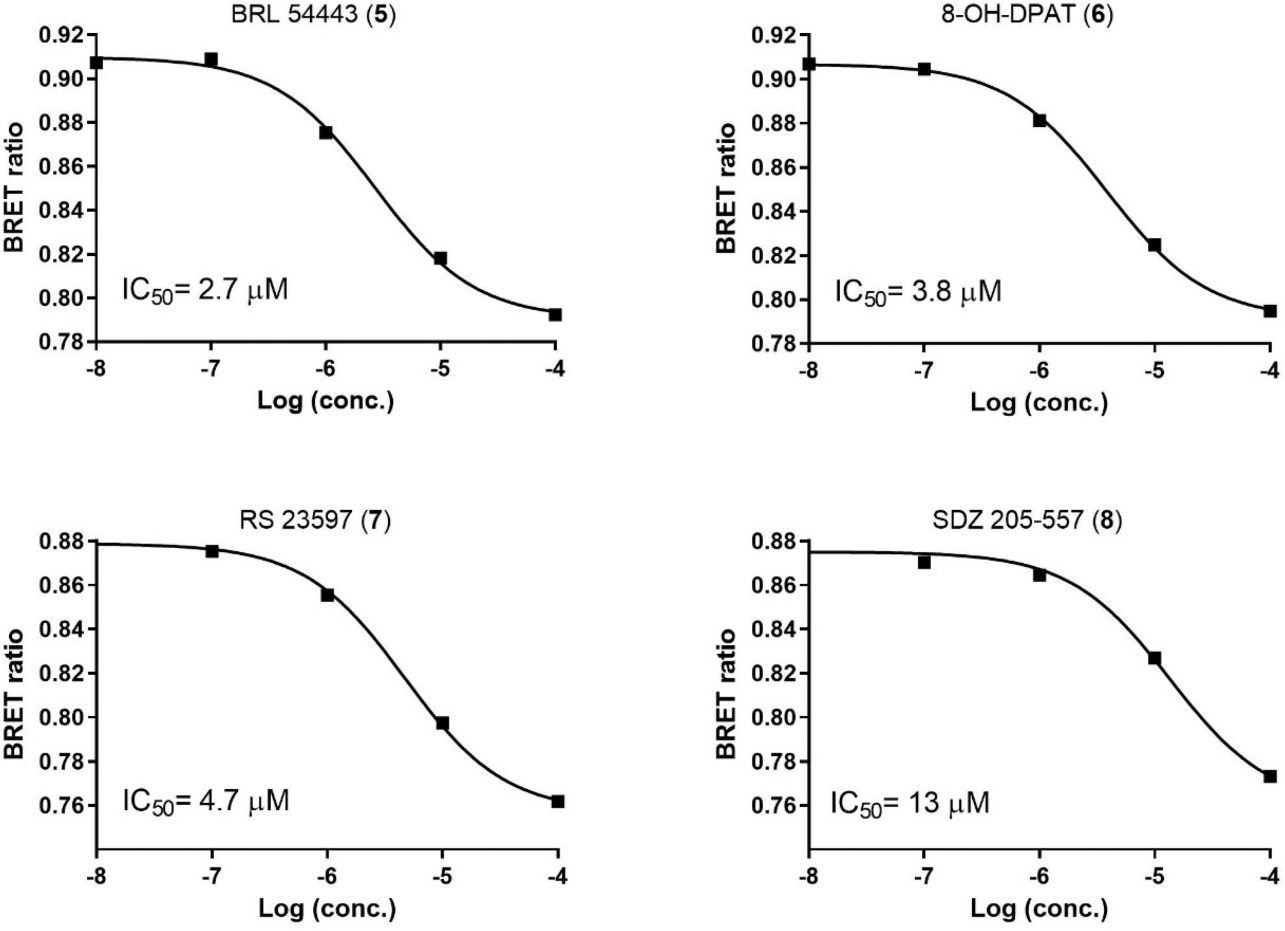
Concentration–response curve of mTAAR5 antagonists **5**–**8**. HEK-293 cells were treated with the compounds at different concentrations, and the BRET ratio was calculated as reflection of cAMP levels (as described in Methods). Data are plotted as concentration–response experiments. Nonlinear regression with one site-specific binding is used to draw the curve using GraphPad Prism9. The data are calculated as mean ± SEM of 3 independent experiments for compounds **5**, **6**, **7**, and **8**.

The set of published mTAAR5 ligands includes agonists, namely trimethylamine (**1**) and alpha-NETA (**2**), as well as antagonists, namely 1-[(5,5-diphenyloxolan-2-yl)methyl]-4-(2-methoxyphenyl)piperazine (**3**) and *N*-(2,2-diphenyl-1,3-dioxolan-4-yl)methyl)-2-(2-methoxyphenoxy)ethan-1-amine (**4**). To enrich the size of the data set, we selected an additional four mTAAR5 antagonists by an in vitro screening of a serotonergic library (Figure 1).

This allowed us to characterize four new antagonists for mTAAR5 (**5**, **6**, **7**, **8**), bringing the number of known mTAAR5 ligands to eight (Table 1). All these tested antagonists show 100% of efficacy (at 100 μM) in blocking mTAAR5 activation by TMA.

**Table 1:**
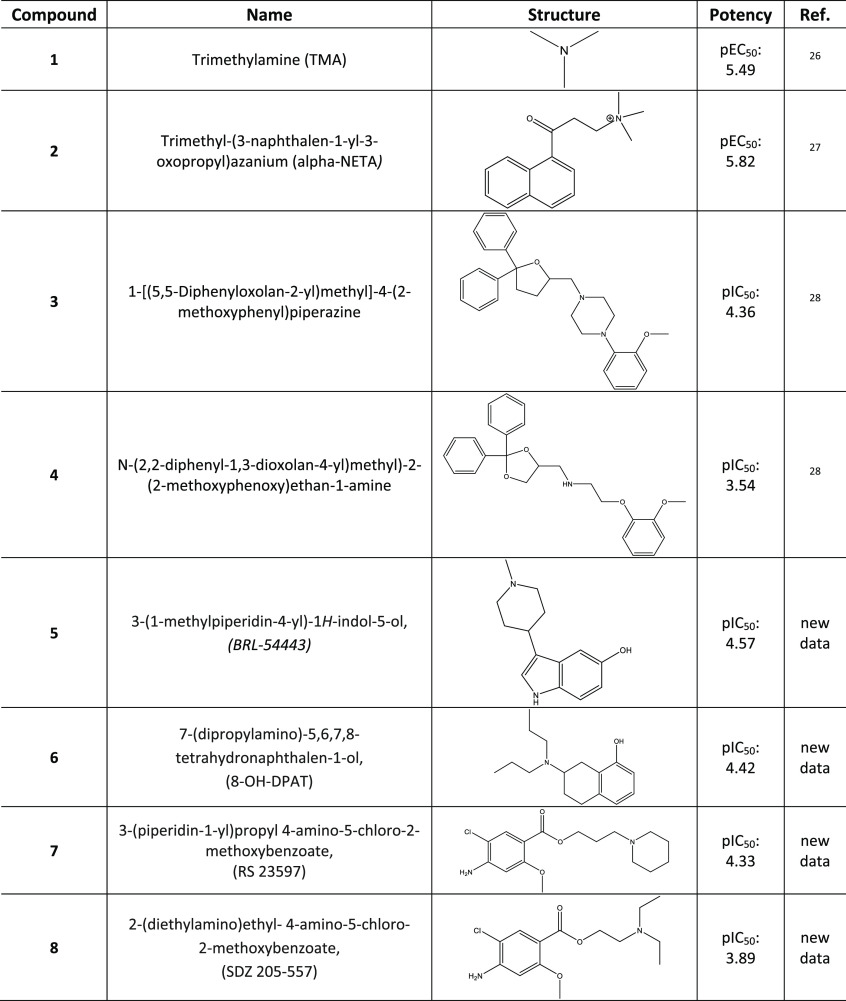
Names, Structures, and Activity (in Modulating cAMP Levels) Values (Indicated as the Negative Log of EC_50_ or IC_50_) of mTAAR5 Ligands; Compounds **5**–**8** Were Tested in This Work

### mTAAR5 Binding Site: Residue Composition

The 3D structure of mTAAR5 was built by using a homology modeling protocol. The wild turkey β1 adrenergic receptor (β1-AR) and human β2 adrenergic receptor (β2-AR) structures in their inactive states (PDB IDs: 2Y03 and 4GBR, respectively) were used as templates. The choice of the conformational state originates from the previous and successful usage of inactive state structures of the class A GPCRs to screen both agonists and antagonists.^32,35,36^ The region of extracellular loop 2 (ECL2) was modeled using the neuropeptide Y1 receptor (PDB ID: 5ZBH). Throughout the manuscript, we consistently apply the structure-based residue numbering system from GPCRdb for class A GPCRs.^37^

**Figure 2:**
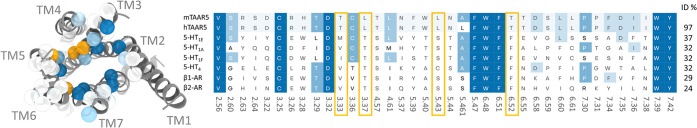
TM binding site sequence alignment of the mouse and human TAAR5, β-ARs, 5-HT_1a_, 5-HT_1e_, 5-HT_1f_ and 5-HT_4_ (receptors that share the ligand space of mTAAR5). The alignment is colored by a gradient scale that transitions from white to blue with the shade becoming darker as the similarity increases. TAAR5-specific residues are highlighted in orange. Conserved and TAAR5-specific residues are mapped on the 3D structure on the left side of the figure. The sequence alignment including all human TAARs is reported in Figure S2.

Interestingly, mTAAR5 antagonists **3**–**8** are known modulators of serotonin receptors 5-HT_1A_, 5-HT_1E_, 5-HT_1F_, and 5-HT_4_ (Table S1). Because of the ligand spectrum overlap, the binding site sequences of mTAAR5 and the cognate serotonin receptors were compared. In Figure 2, we report the sequence alignment of the transmembrane (TM) binding site residues of mouse and human TAAR5 alongside the template used for the homology modeling (β-ARs) and the 5-hydroxytryptamine receptors for which the ligand overlap was found. Interestingly, while β-ARs share the highest similarity when considering full sequences (35% with β1-AR and 30% with the β2-AR, Figure S1), serotonin receptors’ similarity is higher when zooming-in on the binding site, with the highest sequence identity of 37% for 5-HT_1E_.

Like aminergic GPCRs,^38,39^ mTAAR5 also features a conserved aspartate residue in position 3.32. This position is conserved in mouse TAARs, except for the TAAR7a where the aspartate is replaced by glutamate,^40^ and was found to be important for the recognition of trace amines in several TAAR orthologs.^10,23^ In addition to D114^3.32^, residues V87^2.56^, C107^3.25^, F208^5.47^, W265^6.48^, F268^6.51^, W292^7.39^, and Y295^7.42^ are also TM conserved positions among the analyzed GPCRs (Figure 2). C107^3.25^ and C192^45.50^ are part of the highly conserved disulfide bridge of class A GPCRs.^41^ The comparison of binding site sequences revealed also TAAR5-specific positions (T115^3.33^, L119^3.37^, L203^5.43^, and T269^6.52^) that might contribute to the receptor selectivity. At position 3.33, in close proximity with D114^3.32^, TAAR5 has the polar residue T115 replacing an aliphatic residue in serotonin receptors and β-ARs, while, vice versa, in position 3.37, TAAR5 has a leucine instead of a conserved polar residue in serotonin receptors and β-ARs.

Additionally, TAAR5 presents a hydrophobic residue at position 5.43, where β-ARs and 5-HTRs generally have a polar residue. This position is known to be particularly important for ligand binding in class A GPCRs.^42^ Interestingly, TAAR6, TAAR8, and the fish TAAR13c possess an aspartate important for ligand binding of diamine compounds in this position.^43^ One of the most evident differences is in position 6.52, where TAAR5 has a polar threonine, while β-adrenergic and serotonin receptors have a conserved phenylalanine. Experiments utilizing site-directed mutagenesis to modify this specific position in other GPCR members have shown that it not only negatively impacts ligand binding but may also play a role in the receptor’s activation.^44−47^

### mTAAR5 Binding Site: Sampling of Side Chain Orientations

To evaluate the quality of the binding site models generated with homology modeling, we tested the performance in discriminating binders and nonbinders. Ligands in Table 1, excluding TMA because of its small size, were considered as binders. Inactive molecules most structurally similar to the known ligands were retrieved from previous high throughput screening (HTS) campaigns.^27^ Specifically, molecular features in the ranges present in all active structures (Table S2) were used to filter all inactive molecules, yielding a final set of 14 inactive compounds (Table S3). Notably, some inactive molecules in the training set, such as (+)-UH232, N-[2-(piperidinylamino)ethyl]-4-iodobenzamide and metoclopramide, are structurally very similar to ligands **6**, **7**, and **8**. The complete list of active and inactive compounds used for the training set is available at https://github.com/dipizio/mTAAR5_virtual_screening.

Docking simulations were carried out, and the outcome was analyzed with receiver-operating characteristic (ROC) curves. As previously observed for other chemosensory systems,^48−50^ the structure from homology modeling showed poor performance in distinguishing the ligands from the inactive molecules (ROC curve with an AUC value of 0.38). The refinement of the ligand binding site could be a crucial step in developing structure-based ligand design protocols.^49,51,52^ A possible strategy for binding site optimization is the extensive sampling of the binding site conformational space using known active molecules. In this case, we selected the residues to be refined according to the comparison with serotonin receptors at both the sequence and structural levels. At the sequence level, we identified the TAAR5-specific residues to explore the possible conformations and the conserved residues to get an orientation similar to that observed in the serotonin structures. Moreover, we assumed that due to the high similarity of the binding sites, the ligands possibly share a similar binding mode within mTAAR5 and the serotonin receptors. To evaluate the residues involved in ligand binding, compounds **3**–**8** binding modes within the serotonin receptors were investigated through molecular docking simulations and then compared with published studies.^53−57^ All the docking results are available at https://github.com/dipizio/mTAAR5_virtual_screening. Collectively, we found the following positions mostly involved in ligand interactions: 3.29, 3.32, 3.33, 5.43, 6.51, 6.52, 7.38, 7.42, and position 45.52 in the ECL2 (Figure S3). The obtained ligand-bound conformations of serotonin receptors were structurally aligned with the mTAAR5 starting model to identify residues that would compromise mTAAR5 ligand binding conformations predicted for serotonin receptors. The comparison was focused mainly on mTAAR5 antagonists **3** and **4** since these structures occupy relatively large volumes in the orthosteric binding pocket. Hence, we selected R94^2.63^, L203^5.43^, F287^7.34^, D288^7.35^ and I291^7.38^ residues for the refinement process.

Overall, the combined analysis of the mTAAR5 ligand binding modes within 5-HT receptors and sequence-based binding site analysis of mTAAR5 and serotonin receptors provided insight into which residues should undergo an accurate conformational analysis. Two sets of six (R94^2.63^, L203^5.43^, F287^7.34^, T272^6.55^, D288^7.35^, I291^7.38^) and eleven (R94^2.63^, D114^3.32^, L203^5.43^, F208^5.47^, W265^6.48^, F268^6.51^, T272^6.55^, F287^7.34^, D288^7.35^, I291^7.38^, Y295^7.42^) residues were selected. The larger set comprises residues in the proximity of the first set. Using induced fit docking simulations against all known mTAAR5 ligands, 1491 receptor conformers were generated. Structures were then clustered into 75 groups, followed by the evaluation of the representative structure predictive value for each cluster throughout ROC curve analysis (ROC curves of the cluster representative are reported in Figures S4 and S5). Two receptor models (A and B) having ROC AUC values of 0.71 and 0.69, respectively, were selected for the virtual screening campaign.

**Figure 3:**
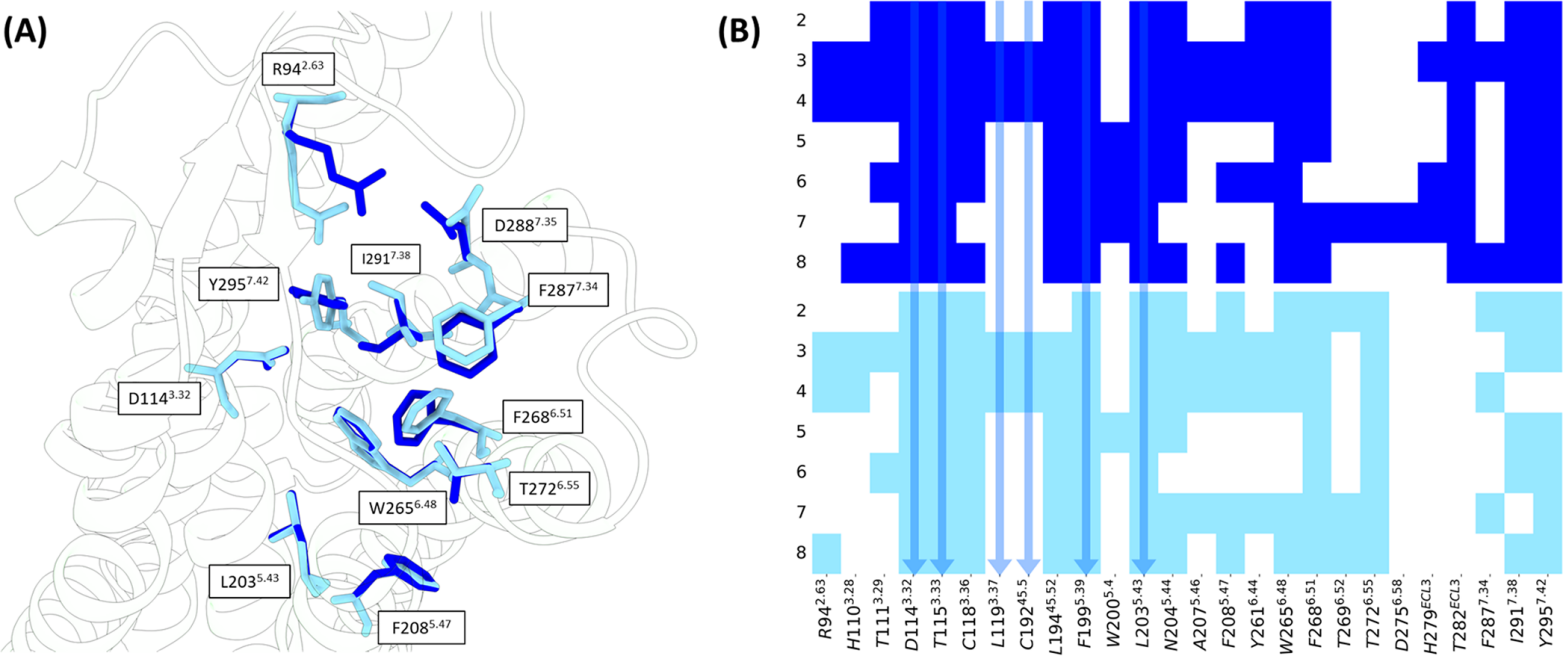
mTAAR5 model A (dark blue) and model B (light blue). (A) 3D representation of the mTAAR5 as cartoon with residues sampled during the simulations in stick. (B) Interaction fingerprints. Colored cells (dark and light blue for model A and model B, respectively) indicate ligand-protein interactions (hydrogen bonds, salt bridges, van der Waals, hydrophobic, π-stacking, and π-cation interactions). Arrows indicate positions where the same patterns of interactions were found by both models. Structures of the binding modes are available at https://github.com/dipizio/mTAAR5_virtual_screening.

The two models were obtained with different induced fit docking settings concerning both the reference ligand and the binding site residues sampled during the simulations. However, the binding site residues show similar conformations in both models (the RMSD of the binding site including the side chains is 1.21 Å), thus suggesting that they reached an overall energetically most favorable conformation. The main structural difference between the selected models lies in the distinct side chain rotameric states observed for R94^2.63^, D288^7.35^, and I291^7.38^ (Figure 3A). These differences slightly affect the binding mode predictions of known ligands, as shown in the interaction fingerprint profiles obtained from the docking of known ligands (compounds **2**–**8**) to the two models (Figure 3B). Interactions observed in most mTAAR5 ligands with both models are an ionic interaction with the conserved D114^3.32^, π–π interactions between the aromatic ring systems present in all mTAAR5 ligands and F268^6.51^. Additionally, compounds **3** and **4** form π–π interactions with conserved W265^6.48^. Although most interactions are captured by both models (D114^3.32^, T115^3.33^, C118^3.36^, L194^45.52^, F199^5.39^, L203^5.43^, N204^5.44^, F268^6.51^, T272^6.55^, I291^7.38^), we can indeed appreciate differences that involve residues with different conformations in the two models (e.g., compounds **3** and **4** form π-charged interactions with R94^2.63^ only in model B), but also affect the binding mode (e.g., compound **7** in model A occupies a different region of the binding pocket close to the ECL3 leading to an ionic interaction with D275^6.58^).

To take into account the potential flexibility of the residues, we performed screening using both receptor models.

### Virtual Screening

**Figure 4:**
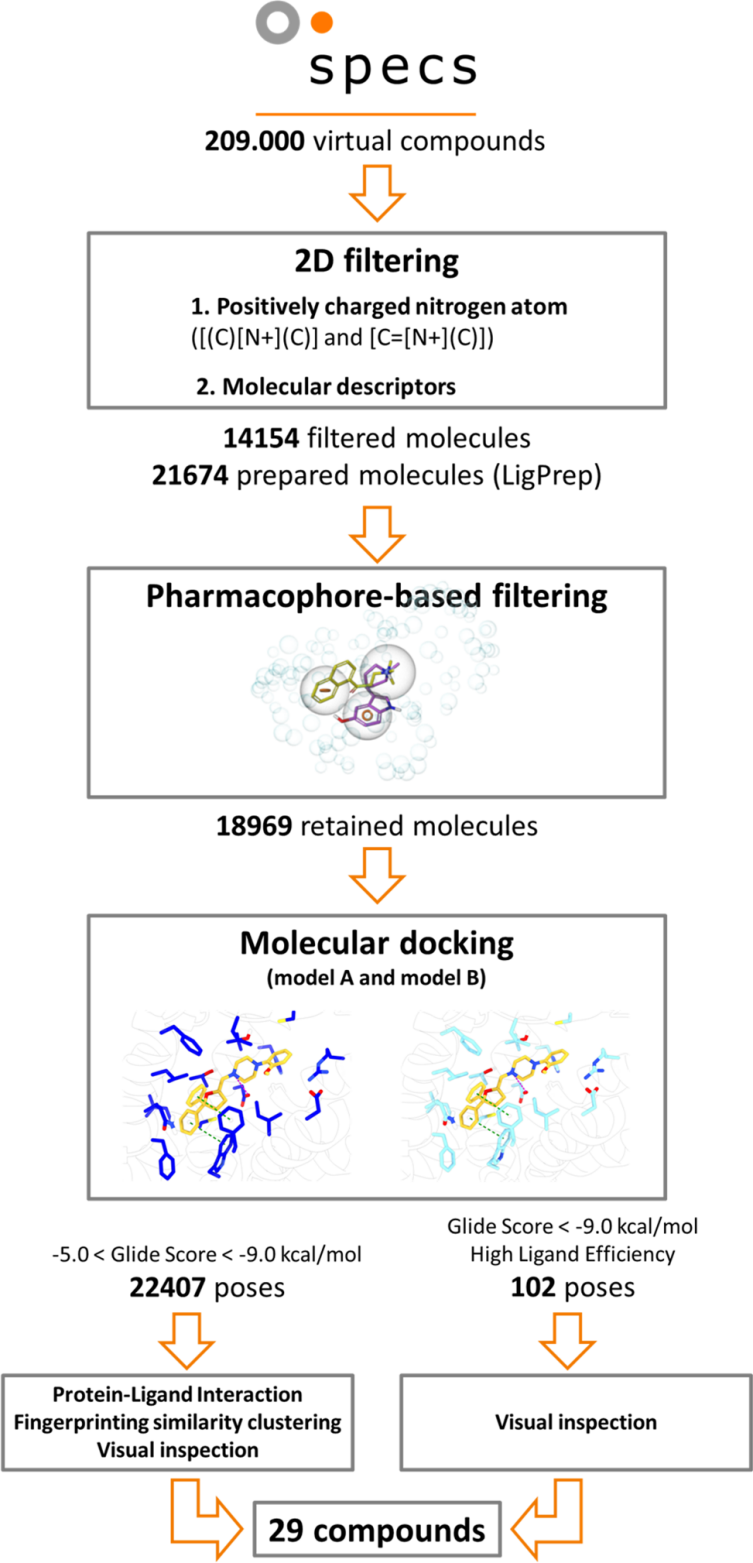
Schematic workflow for virtual screening.

The Specs database screening collection was used as the compound library for virtual screening. Figure 4 shows a schematic of the VS work.

To reduce the docking screening time, the library was filtered by physicochemical properties and pharmacophore features based on the characteristics of the known ligands. We first filtered out compounds lacking a positively charged nitrogen atom, a crucial feature to the formation of a salt bridge with the conserved residue D114^3.32^.^58,59^ Moreover, an additional filter of molecular descriptors is applied (reported in Table S2, we achieved a final reduction of the entire library by 93%, from 209000 to 14154 molecules (Figure 4). The molecules were prepared to generate tautomers and enantiomers and resulted in a set of 21674 molecules.

We then generated a receptor-based pharmacophore hypothesis from the mTAAR5 model, constituted of one positive ionic feature and two aromatic/hydrophobic sites. Indeed, the docking of the most voluminous antagonists **3** and **4** revealed that the diphenyl moiety of both compounds faces the bottom of the orthosteric binding pocket and is stabilized by aromatic stacking interactions with F268^6.51^ and W265^6.48^. Interestingly, these features are mapped individually by other ligands such as **2** and **5**.

The pharmacophore filtering of the database led to a focused screening library of 18969 compounds that was then docked against the mTAAR5 models A and B. The 22407 generated poses with docking scores between −5.5 and −9 kcal/mol were clustered by fingerprint interaction patterns, using the pose of compound **2** as the reference for the similarity matrix. Compound **2** indeed engaged in most of the ligand–receptor interactions shared among all of the compounds (Figure 3B). 940 clusters were generated. Our selection of molecules to be tested was then based on a visual inspection of the cluster representatives and the populations of clusters with desired interaction patterns. We also considered via visual inspections the compounds ranked with the best docking scores (ie, lower than −9.0 kcal/mol), taking into consideration ligand efficiency values. The analysis resulted in the selection of 29 compounds, of which 12 with docking scores ranging between −4.5 and −8.4 kcal/mol were selected from model A and 17 compounds with docking scores between −6.1 and −9.4 kcal/mol from model B (Tables S4 and S5). The complete list with SMILES is available at https://github.com/dipizio/mTAAR5_virtual_screening.

### Newly Identified mTAAR5 Antagonists

TAAR5 is a receptor coupled to a stimulatory G protein, and its activation evokes cAMP production. Therefore, HEK293 cells were cotransfected with mTAAR5 and cAMP BRET biosensor. Light emission changes according to the cAMP fluctuation.^60^ TMA (10 μM) was used as a reference mTAAR5 agonist to evaluate the activity of the compounds throughout the BRET-based assay and tested at 10 μM for either agonistic or antagonistic activity. No compounds displayed an agonistic activity. However, three molecules (compounds **9**, **10**, and **11**) showed mTAAR5 antagonist activity (decreasing cAMP levels induced by TMA) and were therefore subjected to concentration–response assessment with a concentration range from 10 nM to 100 μM. The TMA effect was specific to mTAAR5 activation since no cAMP fluctuation was seen in the absence of mTAAR5 (Figure S6).

**Figure 5:**
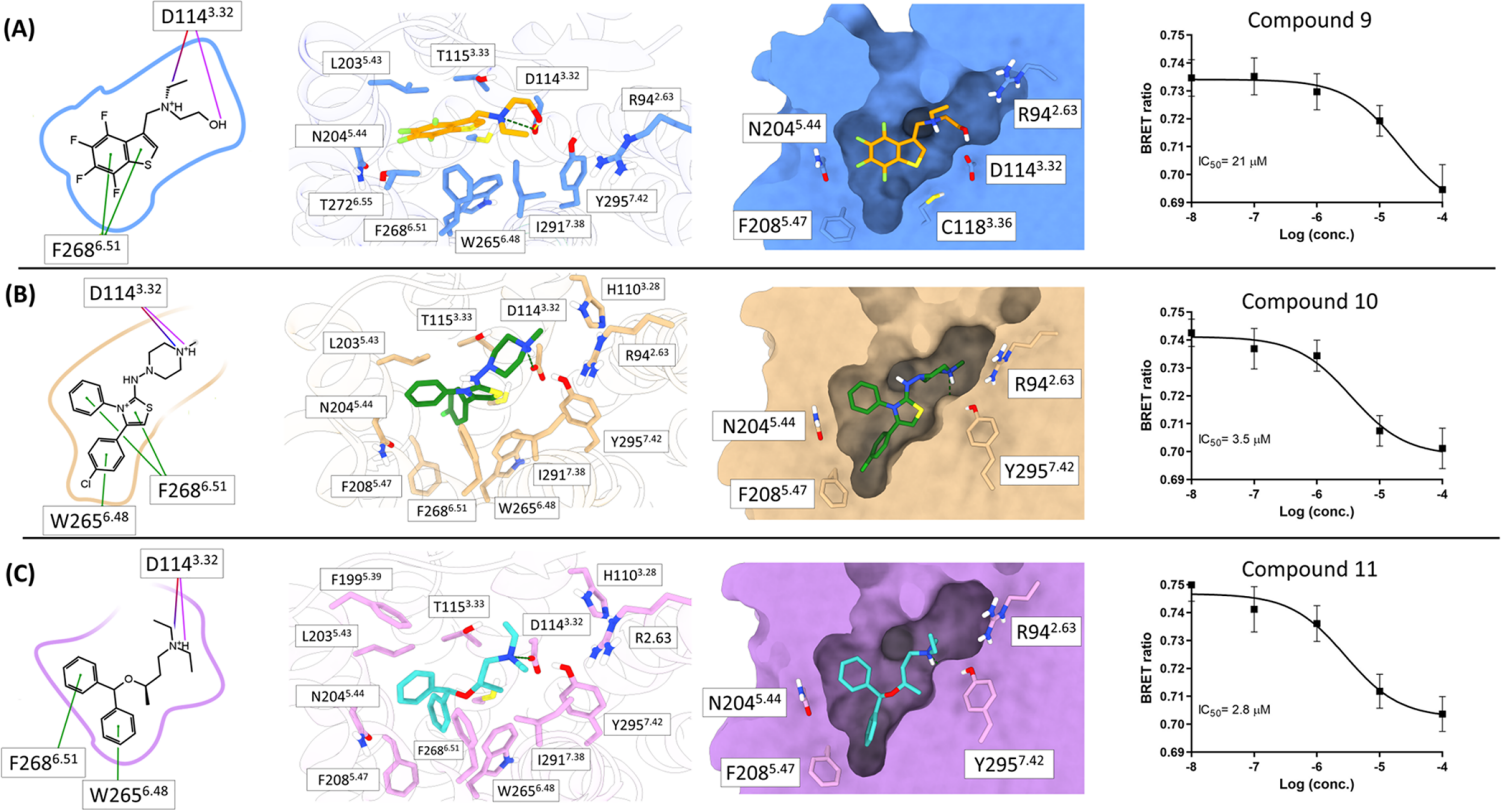
Predicted binding modes and dose-curve responses of compounds **9** (A), **10** (B) and **11** (C). In the first column, we report the 2D structures of the new antagonists and 2D ligand–receptor interactions. Interactions are indicated as follows: salt bridges in violet, hydrogen bonds in magenta, and π–π interactions in green. In the second column, the top-view 3D representation of the binding modes, ligand, and binding site residues are shown as sticks. In the third column, we report the side-view representation of the binding mode, emphasizing the shape of the binding site (only a few residues are shown to provide reference positions in the binding site). Compounds **9**, **10** and **11** are colored in orange, green, and cyan, respectively. mTAAR5 models are colored blue, salmon, and pink when in complex with compounds **9**, **10**, and **11**, respectively. In the last column, we report cAMP variation in cells coexpressing rho-TAAR5 and BRET EPAC biosensor. HEK293 cells were treated with the compounds at different concentrations and plotted as concentration–response experiments. Nonlinear regression with one site-specific binding is used to draw the curve using GraphPad Prism9. The data are plotted as a percentage of inhibition ± SEM of 3 independent experiments for compounds **9**, **10**, and **11**.

Compounds **9**, **10**, and **11** were characterized and structures validated using Ultra High Performance Liquid Chromatography coupled to photo diode array detection (UHPLC-PDA), Ultra High Performance Liquid Chromatography coupled to Time-of-Flight-Mass Spectrometry (UHPLC-ToF-MS), and Nuclear Magnetic Resonance (NMR) experiments (Figure S7). The calculated IC_50_ values of the three compounds **9**, **10**, and **11** were 21 ± 0.18 μM, 3.5 ± 0.15 μM and 2.8 ± 0.16 μM, respectively (Figure 5).

Compound **9** has the lowest ligand efficiency among the three newly discovered antagonists (IC_50_ of 21 μM, selected with model A, docking score of −8.2 kcal/mol, and ligand efficiency of −0.4 kcal/mol, Table S5). It establishes with D114^3.32^ an ionic interaction through the charged aliphatic tertiary amine and a hydrogen bond through the *N′*-ethanol group. The polyfluorinated benzothiophene moiety forms π–π interactions with conserved F268^6.51^ and hydrophobic interactions with L203^5.43^. The *N″*-ethyl group accommodates in a hydrophobic patch formed by the I291^7.38^ and F287^7.34^ (Figure 5A).

Compounds **10** and **11** are bigger than compound **9**, enter deeply into the binding site, and share similar interaction patterns to compounds **3** and **4**.

Compound **10** has the best docking score among the three new antagonists (IC_50_ of 3.5 μM, selected with model B, docking score of −8.6 kcal/mol, ligand efficiency of −0.3 kcal/mol, Table S5). It occupies the deep subpocket enclosed between TM3, 5, and 6 with the chlorophenyl group, where it forms a π–π interaction with the W265^6.48^, a key residue for the class A GPCR activation.^61−63^ It is anchored to the TM6 with two additional π-π interactions with F268^6.51^ (Figure 5B).

Compound **11** is the most potent compound among the three new antagonists (IC_50_ of 2.8 μM, selected with model B, docking score of −7.7 kcal/mol, ligand efficiency of −0.3 kcal/mol, Table S5). It forms an ionic interaction with D114^3.32^ through the charged tertiary amine. As compound **9**, it accommodates the *N′*-ethyl group in the hydrophobic pocket interacting with I291^7.38^ and F287^7.34^. The two aromatic rings establish π- π interactions with both W265^6.48^ and F268^6.51^ (Figure 5C). In both compounds **10** and **11**, the two phenyl groups also have hydrophobic interactions with L203^5.43^, N204^5.44^, and T115^3.33^ and with F208^5.47^ at the bottom of the cavity.

The docking poses of **9**, **10**, and **11** into mTAAR5 were subjected to postdocking Molecular Dynamics (MD) simulations (three replicas for 200 ns for each system, Table S6). Figures S8 and S9 show the RMSD plots of the ligands over the simulation time and the residues most frequently involved in interactions with the three novel antagonists. Importantly, we could notice that ligand poses are stable during the simulation time, implying a potentially correct docking prediction. Very recently, the CryoEM structures of mTAAR9 were published.^64^ The refined binding site of our models is very similar to that of the experimental structures (Figure S10), providing compelling evidence of the robustness of our predictions.

## Discussion

This work led to the identification of seven new mTAAR5 antagonists. We report a virtual screening campaign with a hit rate of 10% with three novel ligands among the 29 experimentally tested molecules. Thus, it represents an achievement in establishing an applicable virtual screening protocol that is especially remarkable as we use a homology model in our campaign. The workflow can therefore be applied to deorphanize other TAARs and unexplored GPCRs for which the experimental structure is not known. We believe that the success of the screening relies on the accurate refinement of the binding site through the comparison with serotonin receptors. Indeed, sampling the binding site conformational space was a required step to generate a receptor conformation that can discriminate between ligands and inactive molecules. Interestingly, the binding site comparison herein described contributes to support the hypothesis that the ancestral ligand of the TAAR family is serotonin, as the TAAR family originated as a duplication of a serotonin receptor.^65^

The discovered antagonists represent novel chemotypes (Table S7 shows the structural similarity of **9**, **10**, and **11** computed against mTAAR5 ligands in the initial data set). The newly identified antagonists and the prediction of their binding poses pinpoint key residues in the binding sites. All three novel antagonists interact with conserved D114^3.32^ and F268^6.51^. The benzyl ring, present in both compounds **10** and **11**, is placed deeply in the binding pocket and can establish additional π–π interactions with W265^6.48^, also known as an activation toggle switch.^58,66,67^ Altogether, the comprehensive structural analysis of the mTAAR5 binding site and the newly discovered antagonists provide the basis for the rational design of potent mTAAR5 antagonists for drug design campaigns.

## Methods

### mTAAR5 Ligand Data

The ligand set was made of one agonist (**2**, alpha-NETA, pEC_50_: 5.82) and six antagonists (**3**, pIC_50_: 4.36; **4**, pIC_50_: 3.54; **5**, pIC_50_: 4.57; **6**, pIC_50_: 4.42; **7**, pIC_50_: 4.33; **8**, pIC_50_: 3.89). The set of inactive molecules was filtered from molecules of the commercially available library Enzo Life Sciences (the SCREEN-WELL neurotransmitter library, 661 compounds) and was inactive in previous HTS campaigns.^27^ Specifically, molecules with the highest similarity to the mTAAR5 ligands were selected using a KNIME workflow.^68^ (i) Duplicates were eliminated. (ii) A KNIME-Maestro Connector node was applied to prepare one 3D structure per entry using the LigPrep.^69^ (iii) The Schrödinger Similarity Matrix node was used to calculate a pairwise distance matrix for the active molecules compared to the inactive ones from unscaled linear, Daylight type fingerprints with a 32-bit precision applying the Tanimoto metrics. Inactive compounds exceeding the distance value cutoff of 0.1 were extracted from the matrix and grouped based on their similarity to the potent molecules. (iv) We then clustered inactive molecules using the Schrödinger Hierarchical Clustering node (hierarchical clustering, average linkage metric, the number of clusters determined by the Kelley index) and the compounds closest to the centroid of each cluster. (v) A final level of filtering was based on the molecular descriptors characterizing the mTAAR5 ligands: 230 < molecular weight < 500, HBD < 5, HBA < 6, TPSA < 140, 0.0 < ALogP < 5.0, number of rotatable bonds < 10, number of chiral centers ≤ 3. Molecules of the active and inactive sets were prepared for docking with LigPrep^69^ setting the maximum number of stereoisomers to be computed to 32 under the retention of specific chirality.

### Homology Modeling

A sequence alignment of mTAAR5 against class A experimental structures was performed with the receptor BLAST tool provided by GPCRdb^70^ to identify experimental structures with the highest sequence identity to mTAAR5. With a sequence identity of 28% and 31%, the crystal structures of the human β_2_-adrenoreceptor (PDB ID: 4GBR) and the wild turkey β_1_-adrenoreceptor (PDB ID: 2Y03) were used to model mTAAR5 structures using MODELER version 9.25.^71^ One hundred structures were generated, and the model with the lowest discrete optimized protein energy (DOPE) score was selected for the following optimization steps. The ECL2 sequence of mTAAR5 is highly diverse from those of the selected templates. The ECL2 of neuropeptide receptor Y1 (PDB ID: 5ZBH) was selected as the template for this region (15% sequence identity). The receptor model was then prepared by optimizing intramolecular hydrogen bonds at physiological pH with the Protein Preparation Wizard in Maestro (Schrödinger Release 2021-3; Maestro, Schrödinger, LLC: New York, NY, 2021). The quality of the receptor model was assessed by the means of different metrics including Ramachandran plot, deviation of bond angles and lengths, steric clashes, side-chain dihedral angles and side-chain planarity, and the DOPE score as implemented in MODELER v9.25.^72,73^ The orthosteric binding site was characterized with SiteMap,^74^ using default parameters. Residues at 3.0 Å of the SiteMap grid points are indicated as binding site residues.

### Binding Modes of mTAAR5 Ligands into 5-HT_1A_, 5-HT_1E_, 5-HT_1F_ and 5-HT_4_ Receptors

Compounds **3**, **4** and **6** were docked into the human 5-HT_1A_ receptor, compound **5** was docked into the human 5-HT_1E_ and 5-HT_1F_ receptors, and compounds **7** and **8** were docked into the human 5-HT_4_ receptor. For these docking simulations, homology models of 5-HT_1A_, 5-HT_1E_, 5-HT_1F_, and 5-HT_4_ were retrieved from SwissModel, GPCRdb^70^ and RosettaGPCR,^75^ respectively. The cross-docking of the compounds into all models of the cognate receptors led to the selection of the homology models and docking settings. Docking poses were compared and evaluated with published binding poses in the literature.^53−56^ The proposed binding mode of compound **5** within the 5-HT_1E_ receptor was confirmed through the X-ray structure released after our modeling work.^54^ Interaction fingerprints were computed with the interaction_fingerprints.py (available at the https://www.schrodinger.com/scriptcenter) using default settings.^76^

The orthosteric binding site in each of the homology models was identified through the Schrödinger SiteMap^74^ using the default parameters. The resulting site points served as input to calculate the grid centroid using the Receptor Grid Generation, Schrödinger Release 2021–3: Glide, Schrödinger, LLC, New York, NY, 2021).^77,78^ Interaction with the conserved residue D114^3.32^ was set as a constraint in the case of the 5-HT_1A_ and 5-HT_1E_ receptors.

### mTAAR5 Binding Site Refinement

The Schrödinger Glide Induced Fit docking protocol (Schrödinger Release 2021–3: Induced Fit Docking protocol; Glide, Schrödinger, LLC, New York, NY, 2021; Prime, Schrödinger, LLC, New York, NY, 2021)^79,80^ was applied to sample the residue conformations within the mTAAR5 binding site. Residues to be sampled were defined according to the 2D and 3D comparison of the binding sites of mTAAR5 with 5-HT_1A_, 5-HT_1E_ and 5-HT_1F_ receptors. Two sets that comprise 6 (R94^2.64^, L203^5.43^, D288^7.35^, F287^7.34^, and I291^7.38^) and 11 (R94^2.64^, D114^3.32^, L203^5.43^, F208^5.47^, T269^6.52^, D288^7.35^, F287^7.34^, W265^6.48^, F268^6.51^, I291^7.38^, Y295^7.42^) residues were selected. Compounds **2**, **3**, **4**, **5**, **6**, **7**, and **8** were docked. In total, 1491 mTAAR5 binding poses were obtained that were clustered using the Schrödinger Conformer Clustering tool (average linkage hierarchical clustering) according to the conformation of the refined residues. The representative structures of each cluster (i.e., the nearest structures to the centroids) were extracted and evaluated for their ability to discriminate active from inactive compounds of the training set. Receptor grids of each receptor conformation were generated on the respective ligand–receptor complexes before docking was performed using the Glide standard protocol (Schrödinger Release 2021–3: Glide, Schrödinger, LLC, New York, NY, 2021).^77,78^ An in-house Python script based on Scikit-learn (v0.24.2) package was used for the ROC curve analysis,^81^ and the data were plotted with Matplotlib Python library.^82^ AUC of the training library was used to evaluate the performance of each model in discriminating between active and inactive compounds. The ROC curves were obtained plotting false positive rate (FPR) vs true positive rate (TPR).

TPR and FPR values are calculated by the following equations:
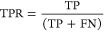
where TP is the number of true positive compounds, and FN is the number of false negative compounds.
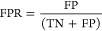
where FP is the number of false positive compounds, and TN is the number of true negative compounds.

Once the most predictive representative structures were identified, the same method was applied to investigate all structures constituting the corresponding clusters. On the basis of this analysis, receptor models A and B were selected for subsequent application in a structure-based virtual screening.

### Interaction Fingerprints and Receptor-Based Pharmacophore Modeling

Interaction fingerprints were computed with the interaction_fingerprints.py (available at the https://www.schrodinger.com/scriptcenter) using the default settings.^76^

The analysis of the docking poses on the refined models highlighted common interactions among the ligands, i.e., a salt bridge with D114^3.32^, aromatic interactions with the residues W265^6.48^ and F268^6.51^. This information was employed to generate a three-feature pharmacophore hypothesis on the receptor–ligand complex of compound **3** and model **4** using the manual method provided by the Schrödinger Phase application.^83^ The pharmacophore model was generated from model B. The positively charged nitrogen atom was selected as positive ionic feature type (*x*: −17.94, *y*: −32.16, *z*: 15.10) and the diphenyl moieties of ligand **3** as two aromatic ring features (*x*: −23.38, *y*: −32.37, *z*: 20.13; *x*: −18.56, *y*: −33.46, *z*: 20.71), each with a match tolerance of 2.0 Å. Additionally, a receptor-based excluded volume shell was created with the default settings. Subsequently, the training set was mapped to the generated pharmacophore for a qualitative evaluation of its performance.

### Virtual Screening Protocol

The Specs screening collection (https://www.specs.net/, 209000 compounds) was chosen as the compound library for the virtual screening campaign. LigPrep^84^ (Schrödinger Release 2021–3: LigPrep, Schrödinger, LLC, New York, NY, 2021) was used for p*K*_a_ calculation and ionization of the molecules at physiological pH (one structure per input molecule was generated). The compounds were filtered according to the presence of a positively charged nitrogen atom (first filter: substructural search with SMARTS patterns ([(C)[N+](C)] and [C=[N+](C)]) using the Schrödinger canvasSearch application (Schrödinger Release 2021–3: Canvas, Schrödinger, LLC, New York, NY, 2021),^85,86^ and molecular properties (second filter: 230 < molecular weight <500, HBD < 5, HBA < 6, TPSA < 140, 0.0 < ALogP < 5.0, number of rotatable bonds <10, number of chiral centers ≤3), using the LigFilter tool.^87^

The structures that passed these two filters were prepared with the LigPrep^69^ to generate stereoisomers and tautomeric states of the subjected molecules while keeping the ionization states unmodified. The pharmacophore screening was used as the third filter using Phase (Schrödinger Release 2021–2: Phase, Schrödinger, LLC, New York, NY, 2021).^71,88^ 250 conformers were generated per compound, each molecule was required to match a minimum of 2 out of 3 pharmacophore sites, the positive feature was set as a required feature to be mapped, and for the aromatic sites both aromatic and hydrophobic features were permitted for the matching. The filtered database was then docked into the two mTAAR5 models (model A and model B) using the Glide standard protocol (Schrödinger Release 2021–3: Glide, Schrödinger, LLC, New York, NY, 2021).^77,78^ The centers of the Glide docking grids were specified by alpha-NETA docked into model A and compound **3** docked into model B.

Top ranked molecules (docking scores −9.0 kcal/mol or lower, Figure 4) and compounds with the highest values of ligand efficiency were visually inspected. The selection was based on ligand–receptor interactions, shape complementarity to the receptor model, and the formation of interactions with mTAAR5 specific residues. Docking poses with scores between −5.5 and −9.0 kcal/mol (Figure 4) were clustered by interaction fingerprints calculated with the poseviewer_interactions.py from Schrödinger (https://www.schrodinger.com/scriptcenter).^76,89^

Tanimoto similarity matrices were generated with respect to the docking poses of alpha-NETA within models A and B and were followed by hierarchical cluster analysis. The resulting representatives and top-ranked molecules of each cluster group were evaluated by visual inspection.

Rendering of the docking poses was done with ChimeraX (v1.3).^90^

### Molecular Dynamics Simulations

We performed postdocking MD simulations of the mTAAR5 model in complex with three new antagonists **9**, **10** and **11** (Table S6). First, we prepared the systems. The Homolwat Web server^91^ was used to add water molecules within the receptor structures, applying settings described in the GPCRmd protocol.^92^ A sodium atom was placed in the allosteric pocket close to the D80^2.50^ (negatively charged state), which proved to be important in stabilizing the inactive state of class A GPCRs. The orientation of the prepared complexes within the membrane bilayer was determined based on the coordinates of the 5-HT1E receptor (PDB ID: 7E33) available in the Orientations of Proteins in Membranes (OPM) database.^93^ Then, the three complexes were superimposed on the OPM reference structure. The prepared complexes were then embedded into a prebuilt 86 Å × 86 Å (with VMD Membrane Builder plugin 1.1) 1-palmitoyl-2oleyl-*sn*-glycerol-3-phospho-choline (POPC) square bilayer through an insertion method^94^ by using HTMD^95^ (Acellera, version 2.2.7). Lipids overlapping with protein residues were removed. TIP3P^96^ water molecules were added to the 86 Å × 86 Å × 116 Å simulation boxes by using VMD Solvate plugin 1.5. The overall charge neutrality was maintained by adding Na^+^/Cl^–^ ions to reach a final physiological concentration of 0.154 M by using VMD Autonize plugin 1.3. All of the N- and C-terminus chains were capped with ACE and CT3, respectively. The CgenFF^97^ (v4.4) and CHARMM36^98,99^ force fields for protein, lipid, TIP3P water model were used for this work. The topology and parameters of the novel antagonists (compound **9**, **10** and **11**) were obtained from the ParamChem Web server (https://cgenff.umaryland.edu/).

ACEMD^100^ (Acellera, version 3.5.1) was used for the MD simulations with periodic boundary conditions. The systems were initially equilibrated through a 3500 conjugate gradient step minimization to reduce clashes induced by the system preparation between protein and lipid/water atoms and then equilibrated with a 100 ns MD simulation in the isothermal–isobaric conditions (NPT ensemble), employing an integration step of 2 fs. The temperature was maintained at 310 K using a Langevin thermostat^101^ with a low damping constant of 1 ps^–1^, and the pressure was maintained at 1.01325 atm using a Montecarlo barostat. Initial restraints of 5 kcal mol^–1^ Å^–2^ were gradually reduced in a multistage procedure over the 100 ns: 5 ns for lipid phosphorus atoms, 60 ns for all protein atoms other than Cα atoms, 80 ns for the protein Cα atoms, and 100 ns for three novel antagonists compound **9**, **10** and **11**. The M-SHAKE algorithm^102^ was used to constrain the bond lengths involving hydrogen atoms. Long-range Columbic interactions were handled using the particle mesh Ewald summation method^103^ with grid size rounded to the approximate integer value of cell wall dimensions. The cutoff distance for long-term interactions was set at 9.0 Å, with a switching function of 7.5 Å. We then computed the membrane thickness using MEMPLUGIN^104^ to evaluate the equilibration stage (38.14 ± 0.64 Å, 37.94 ± 0.32 Å, 37.78 ± 0.35 Å for compound **9**, **10** and **11**, respectively).

We run three independent replicas for each equilibrated system of 200 ns unrestrained MD simulations in the canonical ensemble (NVT) with an integration time step of 4 fs. The temperature was set at 310 K, by setting the damping constant at 0.1 ps^–1^. The root mean square deviation (RMSD) of the backbone carbon alpha and ligand heavy atoms and the contacts between mTAAR5 and three novel antagonists during the MD simulations were computed with an in-house python script based on MDAnalysis (v2.2.0).^105,106^ We used as a reference for the structure alignment the starting mTAAR5 model. For the contact analysis, the three replicas for each system were merged into a single trajectory. The distance cutoff between any atoms of protein and ligand was set to 4.5 Å. Visualization of all data was done with the Matplotlib Python library.^107^

### Cell Culture and Transfection

Human embryonic kidney 293cells (HEK293) were maintained in Dulbecco’s modified Eagle’s, high glucose, GlutaMAX^TM^ medium (Gibco^TM^) supplemented with 10% 331 (v/v) of FBS and 1% penicillin/streptomycin at 37 °C in a humidified atmosphere at 95% air and 5% CO_2_. For the bioluminescence resonance energy transfer (BRET) experiments, cells were plated in 10 cm dishes 24 h prior to the transient transfection of 7 μg of rho-TAAR5 (a generous gift from Prof. Liberles) and 7 μg of EPAC using lipofectamine 2000 (Invitrogen). Five h after transfection, cells were plated in poly-d-lysinecoated 96-well microplates (well-assay white plate with clear bottom, Greiner) at a density of 70,000 cells per well in Opti-MEM (Gibco^TM^) and then cultured for an additional 24 h. The serotonergic ligand library was purchased from Enzo (SCREEN-WELL Serotonergic ligand library).

### BRET Assay

BRET experiments were performed as described previously.^60^ All the compounds were tested at the initial concentration of 10 μM. For the evaluation of the agonistic activity, the plate was read immediately after the addition of the compounds for approximately 20 min. For the evaluation of the antagonistic activity, the compounds were added 5 min before the addition of the control TAAR5 agonist, TMA, and read for approximately 20 min. For the ones that were active, a concentration–response assessment was performed by using different concentrations of the antagonist. TMA is the standard mTAAR5 agonist and induces an increase in cAMP levels. The activity of a putative mTAAR5 antagonist was evaluated in its ability to decrease or abolish the increase of the cAMP levels induced by TMA. The IC_50_ was then calculated by measuring the effect of the compounds against the effect of TMA at 10 μM. Readings were collected using Tecan Infinite instrument that allows the sequential signals integration detected in the 465 to 505 nm and 515 to 555 nm windows. EPAC BRET biosensor was used to monitor cAMP levels. BRET ratio is plotted in the graphs. Increased cAMP specifically reflects an increase in the BRET ratio. The activity of an antagonist was evaluated in terms of the ability to counteract the TMA increase in the BRET ratio. The acceptor/donor ratio was calculated as previously described.^108^ The curve was fitted using nonlinear regression and one site-specific binding with GraphPad Prism 9 software. The data are representative of at least 3 independent experiments and are expressed as means ± SEM.

### Characterization of Compounds **9**, **10** and **11** with UHPLC-PDA

The analyses were carried out using a Shimadzu Nexera XS system, consisting of a SCL-40 system controller, two LC-40D XS pumps, DGU-405 degasser, SIL-40C XS autosampler, CTO-40S column oven, and a SPD-M40 PDA detector (Shimadzu, Duisburg, Germany). Chromatography was done on C18-column (100 × 2.1 mm, 1.7 μ, Kinetex, Phenomenex, Aschaffenburg, Germany). Eluent A was 0.1% formic acid in water, and eluent B was 0.1% formic acid in acetonitrile. After sample injection (1 μL), eluent B was kept at 5% for 2 min and then linearly increased to 100% within 10 min. After 4 min of elution with 100% B, the starting conditions were re-established within 0.5 min and kept 4.5 min for equilibration prior to the next injection. Flow rate was 400 μL/min, the PDA recorded spectra from 190–800 nm. Collected data were investigated with Labsolutions (Shimadzu, Duisburg, Germany).

### Characterization of Compounds **9**, **10** and **11** with UHPLC-ToF-MS

Data acquisition was achieved with a TripleTOF 6600 mass spectrometer (Sciex, Darmstadt, Germany) connected to an ExionLC UHPLC system (Sciex, Darmstadt, Germany). Ionization was detected by positive electrospray (ESI^+^). Ion source parameters were as follows: source temperature 450 °C, curtain gas 35 psi, nebulizer gas 55 psi, turbo gas 65 psi, ion spray voltage 4.5 kV. Accumulation time was 250 ms, declustering potential 80 V, collision energy 10 V, mass range *m*/*z* 50–1000. Chromatography was done on a C18 column (Kinetex C18, 100 × 2.1 mm, 1.7 μm, Phenomenex, Aschaffenburg, Germany) and gradient elution with 0.1% formic acid (mobile phase A) and 0.1% formic acid in acetonitrile (mobile phase B) at a flow rate of 0.25 mL/min, while maintaining a column oven temperature of 40 °C. After injection of the sample (1 μL), eluent B was kept at 5% for 3 min, then increased to 50% within 12 min, then to 100% within 4 min and kept for 2 min. Starting conditions were re-established within 1 min and kept for 5 min prior to the next injection. Collected data were investigated with Peak View 2.2 (Sciex, Darmstadt, Germany).

### Characterization of Compounds **9**, **10** and **11** with NMR

1D and 2D data were recorded on an AV500 NMR spectrometer (500 MHz, Bruker Avance III, Bruker, Rheinstetten, Germany). Solvent was *d*_4_-methanol, and chemical shifts are reported relative to *d*_4_-methanol (^1^H 3.31 ppm, ^13^C 49.00 ppm). Data acquisition and processing were done with Topspin software (versions 3.1 and 4.0, Bruker, Rheinstetten, Germany) and MestReNova software (version 14.1.2; Mestrelab Research S.L., Santiago de Compostella, Spain). Numbering of carbons is according to the structures reported in Figure S7.

## Data Availability

Compounds, mTAAR5 models and predicted binding modes of analyzed compounds within mTAAR5 and the serotonin receptors (5-HT_1A_, 5-HT_1E_, 5-HT_1F_, and 5-HT_4_) can be downloaded from https://github.com/dipizio/mTAAR5_virtual_screening. MD trajectories and related files (topology, parameter, and coordinates) are available at https://zenodo.org/record/8144114.

## Supporting Information Available

Training set molecules; Sequence alignments; Interaction fingerprint analyses; ROC analyses; Chemical characterization of newly identified mTAAR5 antagonists; Molecules selected from the virtual screening, including docking and ligand efficiency scores; MD analyses (PDF)
