# Supplementary material for: Structure-Based Discovery of Mouse Trace Amine-Associated Receptor 5 Antagonists
Source: J Chem Inf Model. 2023 Oct 17;63(21):6667–80. doi: 10.1021/acs.jcim.3c00755 (PMC10647090; doi:10.1021/acs.jcim.3c00755)
Supplement: Supplementary file 1 — ci3c00755_si_001.pdf [file ci3c00755_si_001.pdf]

Supporting Information

Structure-based Discovery of mouse Trace Amine-Associated Receptor 5 Antagonists

Alessandro Nicoli[1,2#], Verena Weber[1,3,4#], Carlotta Bon[5], Alexandra Steuer[1,2], Stefano Gustincich[5], Raul R. Gainetdinov[6], Roman Lang[1], Stefano Espinoza[5,7]*, Antonella Di Pizio[1,2]*

[1] Leibniz Institute for Food Systems Biology at the Technical University of Munich, 85354 Freising, Germany

[2] Chemoinformatics and Protein Modelling, Department of Molecular Life Sciences, School of Life Sciences, Technical University of Munich, 85354 Freising, Germany

[3] Institute for Advanced Simulations (IAS)-5/Institute for Neuroscience and Medicine (INM)-9, Forschungszentrum Jülich, 52428 Jülich, Germany

[4] Faculty of Mathematics, Computer Science and Natural Sciences, RWTH Aachen, Aachen, 52062, Germany

[5] Istituto Italiano di Tecnologia, 16163 Genova, Italy

[6] Institute of Translational Biomedicine and Saint Petersburg University Hospital, Saint Petersburg State University, Saint Petersburg 199034, Russia

[7] Dipartimento di Scienze della Salute, Università del Piemonte Orientale, 28100 Novara, Italy

# These authors contributed equally

* Correspondence:

a.dipizio.leibniz-lsb@tum.de, Tel.: +498161716516

stefano.espinoza@uniupo.it, Tel.: +390321660596

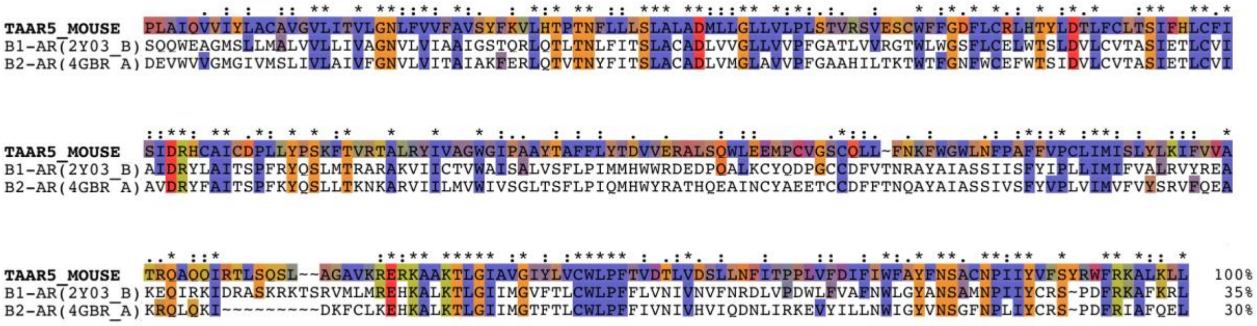

Figure S1. Multiple sequence alignment of the mTAAR5 with the β1- and β2-ARs. Multiple sequence alignment of the mTAAR5 (P30-L319), and the templates used to build the initial homology model: tβ1-AR (4GBR, S37-L356) and hβ2-AR (2YO3, D29-L339). The alignment is colored by residue type. Fully conserved residues are annotated with an ***** (asterisk), a **:** (colon) indicates conservation between groups of strongly similar properties, a **.** (period) indicates conservation between groups of weakly similar properties.

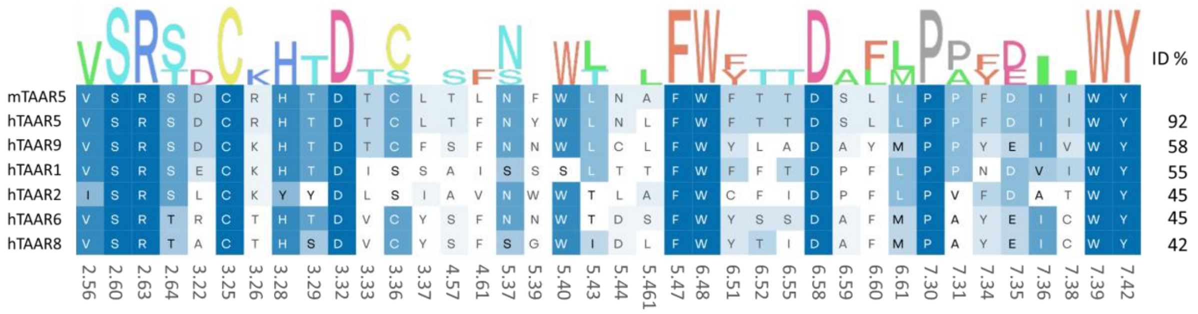

Figure S2. TM binding site multiple sequence alignment of the human TAARs vs. mTAAR5.

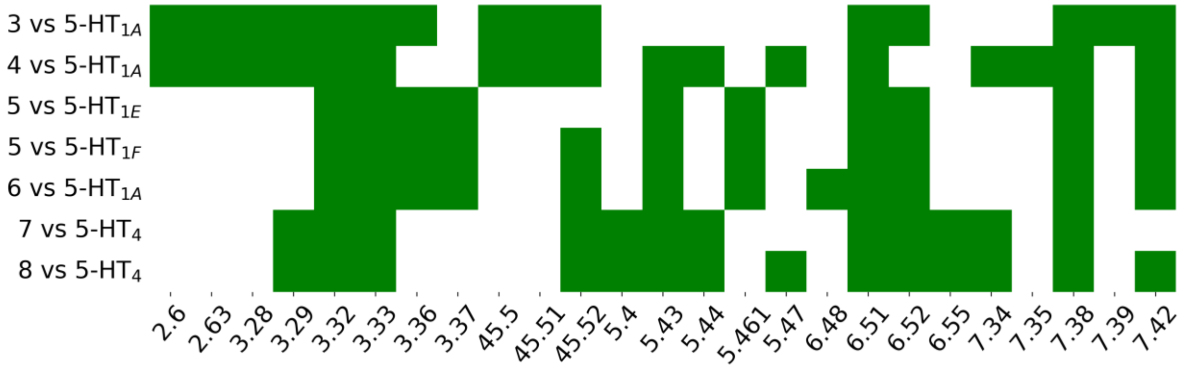

5-HT$_{1A}$	A	Y	F	I	D	V	C	T	C	T	I	T	S	T	A	F	W	F	F	A	G	A	N	W	Y
5-HT$_{1E}$	S	Y	W	L	D	M	C	T	C	T	I	T	S	T	A	F	W	F	F	E	A	D	T	W	Y
5-HT$_{1F}$	S	Y	W	L	D	I	C	T	C	I	I	T	S	T	A	F	W	F	F	E	S	N	A	W	Y
5-HT$_{4}$	G	E	R	T	D	V	T	T	C	V	F	A	C	S	A	F	W	F	F	N	W	T	L	W	Y

Figure S3. Interaction fingerprint analysis of the predicted binding modes of known mTAAR5 ligands 3-8 within the binding site of 5-HT$_{1A}$, 5-HT$_{1E}$, 5-HT$_{1F}$, and 5-HT$_{4}$. Cells are colored white when there is no contact and dark green when the ligand is involved in hydrogen bonds, salt bridges, Van der Waals, hydrophobic, π-stacking, or π-cation interactions with the receptor residues. TM residues are annotated with X.50 GPCR generic residue numbers.[1] Unfortunately, experimental structures of 5-HT$_{1A}$, 5-HT$_{1E}$, 5-HT$_{1F,}$ and 5-HT$_{4}$ receptors were not available at the time of the analysis. Inactive state structure models were retrieved from the GPCRdb[2] (https://gpcrdb.org/), SwissModel[3] (https://swissmodel.expasy.org/) and the RosettaGPCR[4] (https://github.com/benderb1/rosettagpcr) web servers. Crossdocking results were evaluated with the binding poses as described in the literature.[5-9] The selected models used for the interaction fingerprint analysis are reported in the table below: 5-HT$_{1A}$ from GPCRdb for compound **3**; 5-HT$_{1A}$ from RosettaGPCR for compound **4**; 5-HT$_{1E}$ from RosettaGPCR for compound **5**; 5-HT$_{1F}$ from GPCRdb for compound **5**; 5-HT$_{1A}$ from GPCRdb for compound **6**; 5-HT$_{4}$ from RosettaGPCR for compounds **7** and **8.**

Docking poses are available at https://github.com/dipizio/mTAAR5_virtual_screening.

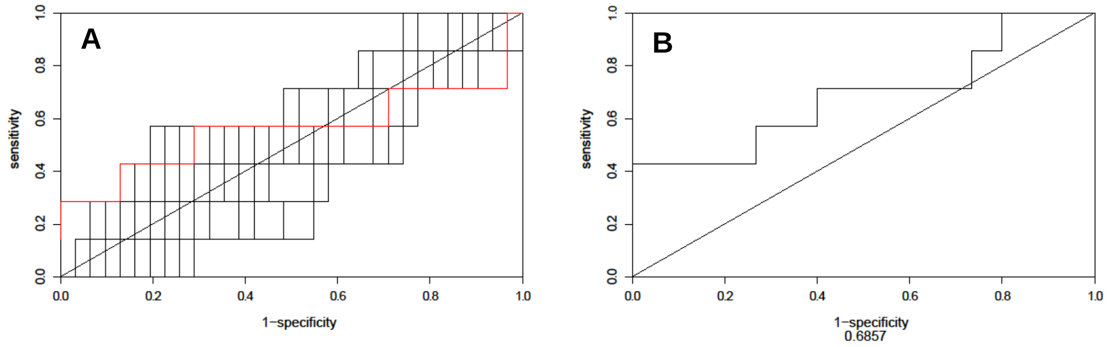

Figure S4. ROC analysis of the 6 residue-subset induced fit docking simulations. (A) ROC curves of the 25 cluster representatives. **(B)** ROC curve of the best model out of cluster 9 (model A).

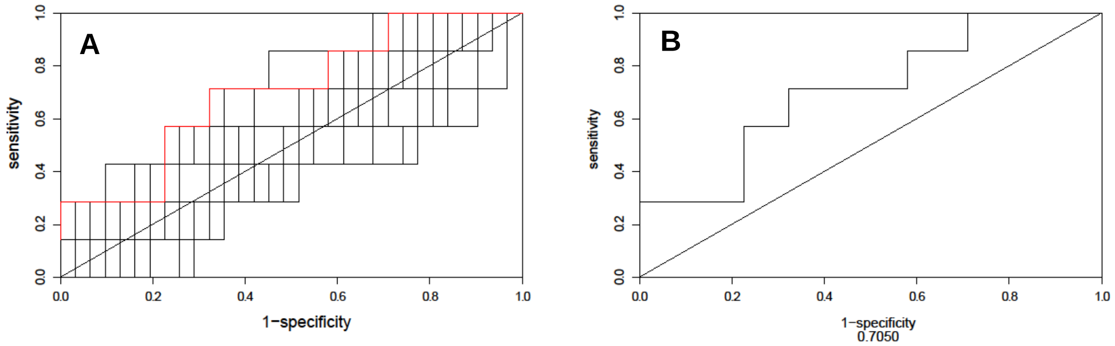

Figure S5. ROC analysis of the 11 residue-subset induced fit docking simulations. (A) ROC curves of the 25 cluster representatives. **(B)** ROC curve of the best model out of cluster 2 (model B).

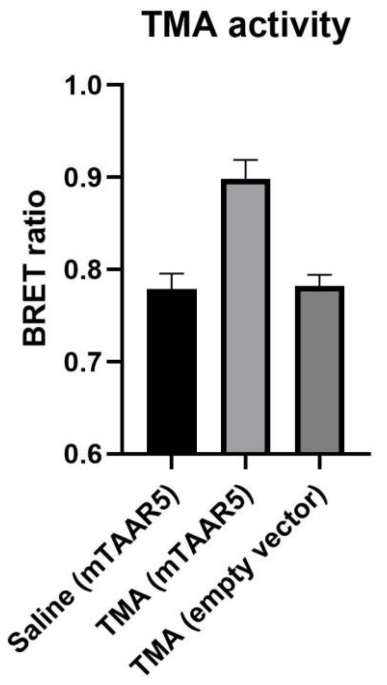

Figure S6. Effect of TMA on cAMP level is mediated by mTAAR5. HEK-293 cells were transfected with EPAC cAMP BRET biosensor with mTAAR5 or empty vector. TMA increases cAMP levels compared to saline only when mTAAR5 is expressed in the cells but not in empty vector CTRL cells. The data are calculated as mean ± SEM of 3 independent experiments.

Compound 9 (AA-516/31408053)

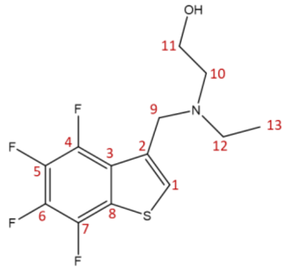

UHPLC-PDA: Rt. 5.82 min, λ_{max} 295, 216, 196 nm; UHPLC-TOF-MS: Rt. 10.41 min, *m/z* 308.0756, calculated 308.0727 (Δ+9.5 ppm) for $C_{13}H_{13}F_4NOS$ ([M+H]$^+$); ^{13}C (δ ppm) 8.9 (CH_3, C13), 49.7 (CH_2, C12), 51.6 (CH_2, C9, J_{CF} =5.4 Hz), 55.1 (CH_2, C10), 56.6 (CH_2, C11), 124.8 (C, C2), 124.9 (C, C8, dd, J_{CF} =13.9, 2.7 Hz), 125.6 (C, C3, ddd, J_{CF} =17.4, 5.8, 2.2 Hz), 136.6 (CH, C1), 138.8 (CF, C5, ddd, J_{CF} =100.6, 14.5, 2.6 Hz), 140.5 (CF, C6, ddd, J_{CF} =98.2, 15.6, 2.2 Hz), 142.8 (CF, C7, m), 144.4 (CF, C4, m); ^{1}H (δ ppm) 1.43 (3H, t, J=7.3 Hz, H13), 3.39 (2×2H, q, J=7.3 Hz, H10, H12), 3.95 (2H t, J=3.9 Hz, H11), 4.72 – 4.86 (H9, deduced from HSQC), 8.20 (3H, s, H1).

Compound 10 (AF-399/41085043)

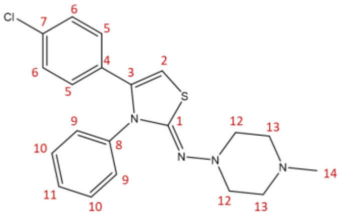

UHPLC-PDA: Rt. 6.01 min, λ_{max} 261, 222, 194 nm; UHPLC-TOF-MS: Rt. 10.97 min, *m/z* 385.1273, calculated 385.1248 (Δ+6.4 ppm) for $C_{20}H_{21}ClN_4S$ ([M+H]$^+$); ^{13}C (δ ppm) 45.6 (CH_3, C14), 54.7 (CH_2, C12), 55.9 (CH_2, C13), 102.6 (CH, C2), 129.2 (CH, C11), 129.4 (CH, C5 or C6 or C9 or C10), 129.9 (CH, C5 or C6 or C9 or C10), 130.4 (CH, C5 or C6 or C9 or C10), 131.1 (CH, C5 or C6 or C9 or C10), 131.4 (C, C4), 135.4 (CCl, C7), 138.9 (C, C8), 140.8 (C, C3), 172.2 (C, C1); ^{1}H (δ ppm) 2.33 (3H, s, H14), 2.45 – 2.71 (broadened signal, H13), 2.80 (4H, bs, H12), 6.30 (1H, s, H2), 7.05 – 7.40 (9H, phenyl, H5, H6, H9, H10, H11).

Compound 11 (AG-690/08755015)

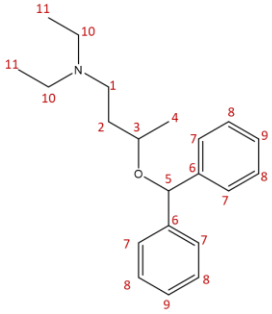

UHPLC-PDA: Rt. 6.97 min, λ_{max} 249, 220, 197 nm; UHPLC-TOF-MS: Rt. 14.34 min, *m/z* 312.2337, calculated 312.2322 (Δ+4.8 ppm) for $C_{21}H_{29}NO$ ([M+H]$^+$); ^{13}C (δ ppm) 9.22 (2×CH_3, C11), 19.9 (CH_3, C4), 31.8 (CH_2, C2), 48.5 (2×CH_2, C10), 49.9 (CH_2, C1), 72.1 (CH, C3), 82.3 (CH, C5), 127.7 (2×CH, C7 or CH, C8), 128.3 (CH, C9), 128.7 (2×CH, C7 or CH, C8), 128.9 (CH, C9), 129.3 (2×CH, C7 or CH, C8), 129.6 (2×CH, C7 or CH, C8), 143.7 (C, C6), 144.3 (C, C6); ^{1}H (δ ppm) 1.24 (2×3H, t, J=7.3 Hz, H11), 1.26 (3H, d, J=6.2 Hz), 1.90 (2H, m, H2), 3.00 (1H, m, H1a), 3.16 (2×2H, q, J=7.4 Hz, H10), 3.23 (1H, m, H1b), 3.70 (1H, m, H3), 5.56 (1H, s, H5), 7.20 – 7.40 (10H, phenyl).

Figure S7. Chemical characterization of HIT compounds 9, 10 and 11 with UHPLC-PDA, UHPLC-TOF-MS, ^{1}H and ^{13}C NMR.

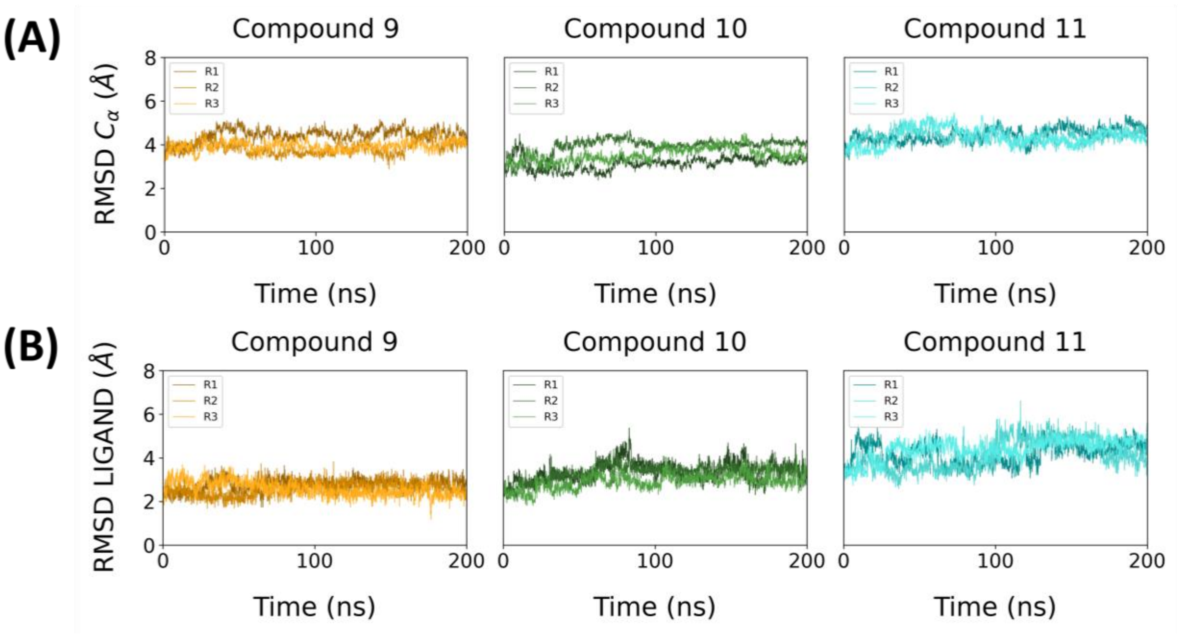

Figure S8. (A) RMSD plots of the alpha carbons of mTAAR5 in complex with compounds **9** (orange line), **10** (greens line) and **11** (blues lines) during the MD simulations. **(B)** RMSD plots of compounds **9, 10 and 11** heavy atoms during the MD simulations.

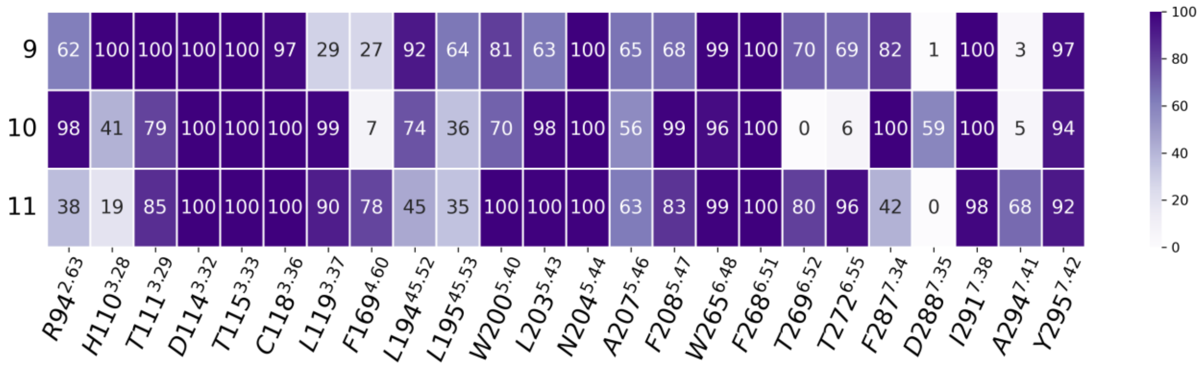

Figure S9. Contact frequencies of 9, 10, and 11 in complex with mTAAR5 along the MD trajectories. Cells are colored in purple shades, darkness of the color increases with increased values of frequencies. The distance cutoff was set to 4.5 Å.

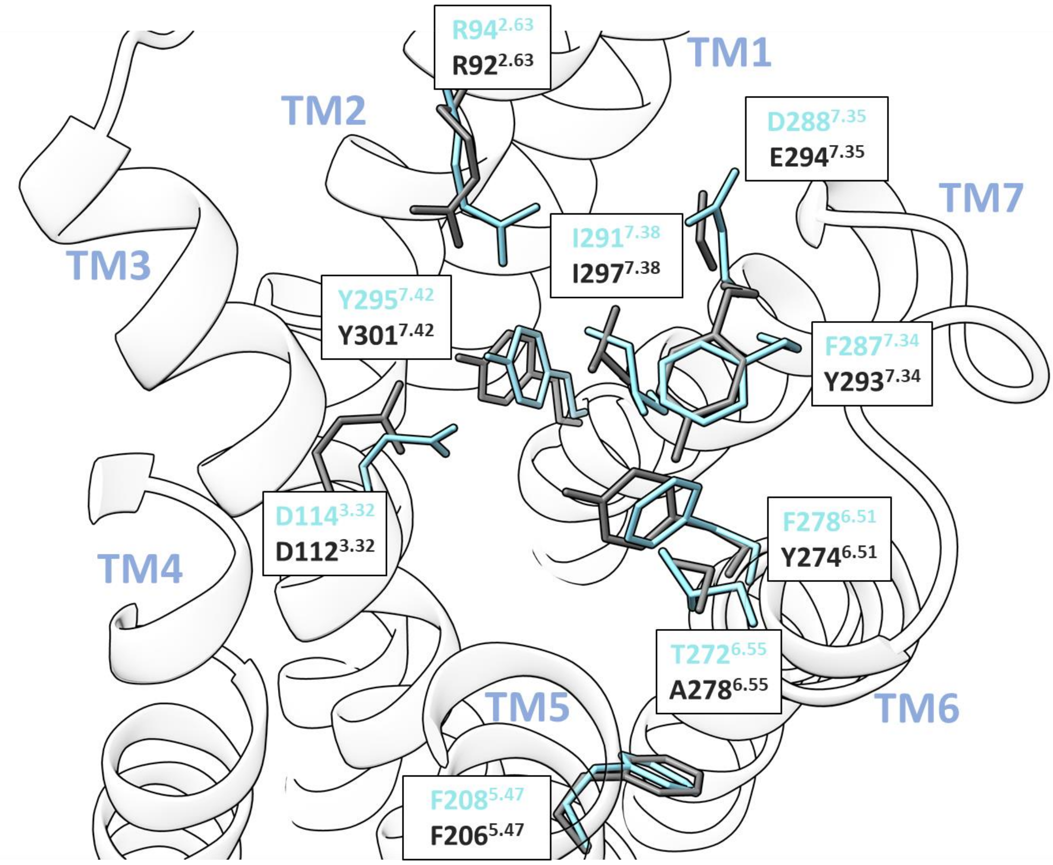

Figure S10. Structural superimposition of model B with the experimental mTAAR9 structure (PDB ID: 8IW7). The receptor backbone is represented as white transparent cartoon. Selected refined residues are shown as cyan and grey licorice for mTAAR5 and mTAAR9, respectively. The position that differs between mTAAR5 and mTAAR9 are 6.51 (F_{mTAAR5}/Y_{mTAAR9}), 6.55 (T_{mTAAR5}/A_{mTAAR9}) and 7.34 (F_{mTAAR5}/Y_{mTAAR9}) and 7.35 (D_{mTAAR5}/E_{mTAAR9}). W265$^{6.48}$ is not resolved in 8IW7 and it is not shown in the structural representation, it adopts an inactive conformation in the mTAAR5 model. Additionally, we have a different position of L203$^{5.43}$ between the mTAAR5 model and the mTAAR9 experimental structure, not shown here for graphical clarity. Interestingly, in mTAAR5, Y295$^{7.42}$ and R94$^{2.63}$ adopt the same rotameric state shown in mTAAR9 after refinement in model B. Notably, the refined model has a similar orientation of the eleven refined residues compared to the mTAAR structure (RMSD of backbone C_α: 1.53 Å).

Table S1. Activation data of mTAAR5 ligands towards serotonin receptors.

Compound	5-HT_{1A}	5-HT_{1E}	5-HT_{1F}	5-HT_4	Ref.
**3**	pK_i: 7.64	-	-	-	[10]
**4**	pK_i: 9.22	-	-	-	[5]
**5**	-	pEC_{50}:8.16	pK_i:8.15	-	[6, 11-12]
**6**	pEC_{50}: 7.24	-	-	-	[13-14]
**7**	-	-	-	pIC_{50}: 6.31	[15-16]
**8**	-	-	-	pIC_{50}: 6.11	[17]

Table S2. Molecular descriptors and common features of the mTAAR5 ligands. MW: molecular weight (g/mol); HBD: hydrogen-bond donor; HBA: hydrogen-bond acceptor; RB: number of rotatable bonds; TPSA: topological polar surface area ($Å^2$). The list of ligands with SMILES is available at https://github.com/dipizio/mTAAR5_virtual_screening.

Compound	MW	ALogP	HBD	HBA	RB	TPSA	nCC
**2**	242.3	-1.7	0	1	4	17	0
**3**	429.6	5.3	0	2	6	25	1
**4**	406.5	4.3	0	4	9	49	1
**5**	231.3	2.0	2	1	1	39	1
**6**	248.4	4.2	1	1	5	23	1
**7**	327.8	2.6	1	3	7	65	0
**8**	301.8	2.3	1	3	8	65	0
**Common molecular features:**							
C[N+]C	230 - 500	0.0 - 5.0	< 5	< 6	< 10	< 140	<= 3

Table S3. Molecular descriptors of the inactive molecules selected for the training set. MW: molecular weight (g/mol); HBD: hydrogen-bond donor; HBA: hydrogen-bond acceptor; RB: number of rotatable bonds; TPSA: topological polar surface area (Å^2); nCC: number of chiral center. The list with SMILES is available at https://github.com/dipizio/mTAAR5_virtual_screening.

Compound	MW	ALogP	HBD	HBA	RB	TPSA	nCC
**N-Butyl-N-Ethyl-2-(1-naphthyloxy)ethanamine**	272.4	3.0	0	1	8	39.1	0
**BMY 7378**	386.5	1.1	0	3	5	46.0	0
**NAN-190**	394.5	1.7	0	5	7	50.5	0
**WB 4101**	346.4	1.6	1	4	8	43.6	1
**ICI 89406**	355.4	1.0	4	2	9	45.3	1
**Alprenolol**	250.4	1.4	2	1	8	33.0	1
**(±)-2-(N-phenylethyl-N-propyl)amino-5-hydroxytetralin**	310.5	3.7	1	1	6	44.7	1
**trans-7-Hydroxy-PIPAT**	372.3	2.4	1	1	5	38.5	1
**Ropinirole**	261.4	1.5	1	2	7	34.5	0
**U-99194A**	278.4	2.5	0	1	7	36.1	0
**(+)-UH 232**	276.4	3.3	0	2	6	37.3	2
**Metoclopramide**	300.8	0.22	2	3	8	35.5	0
**S(-)-raclopride**	348.2	1.5	2	1	6	37.8	1
**N-[2-(piperidinylamino)ethyl]-4-iodobenzamide**	359.2	1.1	1	1	5	35.8	0

Table S4. Molecular descriptors of the compounds selected with virtual screening. MW: molecular weight (g/mol); HBD: hydrogen-bond donor; HBA: hydrogen-bond acceptor; RB: number of rotatable bonds; TPSA: topological polar surface area (Å^2); nCC: number of chiral center. Compound **9**, **10**, **11** are the experimentally validated mTAAR5 antagonists identified in this work. The list with SMILES is available at https://github.com/dipizio/mTAAR5_virtual_screening.

Compound	Specs ID	MW	ALogP	HBA	HBD	RB	TPSA	nCC
**9**	AA-516/31408053	308.32	1.609	1	1	5	51.71	1
**10**	AF-399/41085043	386.95	3.544	0	1	4	47.38	0
**11**	AG-690/08755015	312.479	2.962	1	0	9	12.47	1
**12**	AK-968/12115029	299.396	0.275	2	1	5	41.57	0
**13**	AN-740/37278024	307.826	0.659	1	2	4	39.6	0
**14**	AE-641/30153046	379.301	3.239	1	0	6	12.47	1
**15**	AE-641/00584062	274.773	2.412	1	0	4	20.31	0
**16**	AE-641/30153045	292.352	2.235	1	0	6	12.47	0
**17**	AT-057/43469073	290.412	0.013	1	2	4	44.53	0
**18**	AE-641/11702964	343.295	3.394	0	0	2	3.24	1
**19**	AI-204/31681037	275.33	0.031	3	1	3	53.01	0
**20**	AA-516/30012006	301.232	1.186	1	1	4	51.71	1
**21**	AK-968/13146376	303.378	2.295	0	0	4	6.48	0
**22**	AO-365/15162001	330.454	2.984	1	0	5	20.31	2
**23**	AK-968/11844162	318.226	0.695	2	1	5	41.57	0
**24**	AG-690/40720074	401.533	2.737	2	0	6	32.78	0
**25**	AS-871/11777022	415.517	2.522	3	0	5	42.01	0
**26**	AM-814/41094406	292.352	2.235	1	0	6	12.47	1
**27**	AG-205/36566049	343.474	3.965	2	1	5	53.98	0
**28**	AG-690/11629181	256.371	1.334	1	1	5	23.47	0
**29**	AG-680/20240028	278.421	3.207	0	0	4	3.24	0
**30**	AK-968/12265016	270.357	0.459	2	1	2	35.58	0
**31**	AA-516/12432063	243.355	0.754	0	1	1	19.37	0
**32**	AE-641/40777758	231.32	1.031	2	1	2	36.36	0
**33**	AO-022/43453354	273.358	0.968	2	0	2	32.78	0
**34**	AG-690/11959690	252.311	1.386	2	0	5	29.54	0
**35**	AE-641/04595048	268.766	1.845	2	0	5	29.54	0
**36**	AE-641/14824009	257.359	1.492	2	1	2	36.36	0
**37**	AO-476/43250130	291.806	1.859	2	0	1	32.26	0

Table S5. Virtual screening selection. Specs ID, selection mode, docking and ligand efficiency (LE) scores of molecules selected with the virtual screening. Only two compounds (**24** and **25**) were selected among the molecules with highest docking scores (> -9.0 kcal/mol), while all the others were selected after the postprocessing of docking poses (interaction fingerprints analyses). Compound **9**, **10**, **11** are the experimentally validated mTAAR5 antagonists identified in this work.

Compound	Specs ID	VS selection	docking score Model A	docking score Model B	LE Model A	LE Model B
**9**	AA-516/31408053	postpresessing	-8.244	-8.652	-0.412	-0.433
**10**	AF-399/41085043	postpresessing	-7.278	-8.568	-0.28	-0.33
**11**	AG-690/08755015	postpresessing	-6.742	-7.695	-0.293	-0.335
**12**	AK-968/12115029	postpresessing	-8.271	-7.179	-0.376	-0.326
**13**	AN-740/37278024	postpresessing	-8.12	-7.998	-0.387	-0.381
**14**	AE-641/30153046	postpresessing	-8.03	-8.806	-0.349	-0.383
**15**	AE-641/00584062	postpresessing	-7.928	-7.376	-0.417	-0.388
**16**	AE-641/30153045	postpresessing	-7.917	-8.796	-0.377	-0.419
**17**	AT-057/43469073	postpresessing	-7.731	-7.641	-0.368	-0.364
**18**	AE-641/11702964	postpresessing	-7.549	-8.051	-0.359	-0.383
**19**	AI-204/31681037	postpresessing	-7.353	-7.453	-0.368	-0.373
**20**	AA-516/30012006	postpresessing	-7.222	-7.438	-0.451	-0.465
**21**	AK-968/13146376	postpresessing	-6.516	-6.073	-0.296	-0.276
**22**	AO-365/15162001	postpresessing	-8.248	-7.993	-0.33	-0.32
**23**	AK-968/11844162	postpresessing	-6.89	-6.571	-0.344	-0.329
**24**	AG-690/40720074	docking score	-4.464	-9.417	-0.149	-0.314
**25**	AS-871/11777022	docking score	-8.319	-9.338	-0.268	-0.301
**26**	AM-814/41094406	postpresessing	-7.889	-8.861	-0.376	-0.422
**27**	AG-205/36566049	postpresessing	-8.372	-8.397	-0.335	-0.336
**28**	AG-690/11629181	postpresessing	-5.757	-8.309	-0.303	-0.437
**29**	AG-680/20240028	postpresessing	-6.755	-8.066	-0.322	-0.384
**30**	AK-968/12265016	postpresessing	-6.85	-7.482	-0.343	-0.374
**31**	AA-516/12432063	postpresessing	-7.503	-7.294	-0.417	-0.405
**32**	AE-641/40777758	postpresessing	-7.078	-7.258	-0.416	-0.427
**33**	AO-022/43453354	postpresessing	-6.187	-7.09	-0.309	-0.354
**34**	AG-690/11959690	postpresessing	-5.926	-6.84	-0.329	-0.38
**35**	AE-641/04595048	postpresessing	-6.211	-6.837	-0.345	-0.38
**36**	AE-641/14824009	postpresessing	-6.166	-7.175	-0.325	-0.378
**37**	AO-476/43250130	postpresessing	-5.165	-7.709	-0.258	-0.385

Table S6. List and information of simulated systems. Topology, parameter, and coordinates files as well as MD trajectories are available at https://zenodo.org/record/8144114.

System	Size	Length	Replicas	Total MD Sampling
mTAAR5-Compound **9**	Atoms= 79104<br>Box= 86 Å x 86 Å x 116 Å	200 ns	3	600 ns
mTAAR5-Compound **10**	Atoms= 79105<br>Box= 86 Å x 86 Å x 116 Å	200 ns	3	600 ns
mTAAR5-Compound **11**	Atoms= 79114<br>Box= 86 Å x 86 Å x 116 Å	200 ns	3	600 ns

Table S7. Tanimoto similarity of compounds **9**, **10**, **11** respect to the known mTAAR5/5-HTR ligands in the initial dataset (compounds **2-8**). The Morgan fingerprints, with a radius of 2 and encoded using 2048 bits, were computed using the Python package RDKit.[18] Subsequently, Tanimoto similarity metrics were calculated based on these fingerprints.

	**9**	**10**	**11**
**2**	0.18	0.15	0.15
**3**	0.28	0.30	0.32
**4**	0.22	0.24	0.42
**5**	0.24	0.23	0.15
**6**	0.20	0.21	0.18
**7**	0.23	0.25	0.28
**8**	0.24	0.25	0.22

References

(1) Isberg, V.; de Graaf, C.; Bortolato, A.; Cherezov, V.; Katritch, V.; Marshall, F. H.; Mordalski, S.; Pin, J. P.; Stevens, R. C.; Vriend, G.; Gloriam, D. E., Generic GPCR residue numbers - aligning topology maps while minding the gaps. *Trends Pharmacol Sci* **2015,** *36*, 22-31.

(2) Pandy-Szekeres, G.; Munk, C.; Tsonkov, T. M.; Mordalski, S.; Harpsoe, K.; Hauser, A. S.; Bojarski, A. J.; Gloriam, D. E., GPCRdb in 2018: adding GPCR structure models and ligands. *Nucleic Acids Res* **2018,** *46*, D440-D446.

(3) Waterhouse, A.; Bertoni, M.; Bienert, S.; Studer, G.; Tauriello, G.; Gumienny, R.; Heer, F. T.; de Beer, T. A. P.; Rempfer, C.; Bordoli, L.; Lepore, R.; Schwede, T., SWISS-MODEL: homology modelling of protein structures and complexes. *Nucleic Acids Res* **2018,** *46*, W296-W303.

(4) Bender, B. J.; Marlow, B.; Meiler, J., Improving homology modeling from low-sequence identity templates in Rosetta: A case study in GPCRs. *PLoS Comput Biol* **2020,** *16*, e1007597.

(5) Prandi, A.; Franchini, S.; Manasieva, L. I.; Fossa, P.; Cichero, E.; Marucci, G.; Buccioni, M.; Cilia, A.; Pirona, L.; Brasili, L., Synthesis, biological evaluation, and docking studies of tetrahydrofuran-cyclopentanone- and cyclopentanol-based ligands acting at adrenergic alpha(1)- and serotonine 5-HT1A receptors. *J Med Chem* **2012,** *55*, 23-36.

(6) Xu, P.; Huang, S.; Zhang, H.; Mao, C.; Zhou, X. E.; Cheng, X.; Simon, I. A.; Shen, D. D.; Yen, H. Y.; Robinson, C. V.; Harpsoe, K.; Svensson, B.; Guo, J.; Jiang, H.; Gloriam, D. E.; Melcher, K.; Jiang, Y.; Zhang, Y.; Xu, H. E., Structural insights into the lipid and ligand regulation of serotonin receptors. *Nature* **2021,** *592*, 469-473.

(7) Del Bello, F.; Bonifazi, A.; Giorgioni, G.; Quaglia, W.; Amantini, C.; Morelli, M. B.; Santoni, G.; Battiti, F. O.; Vistoli, G.; Cilia, A.; Piergentili, A., Chemical manipulations on the 1,4-dioxane ring of 5-HT1A receptor agonists lead to antagonists endowed with antitumor activity in prostate cancer cells. *Eur J Med Chem* **2019,** *168*, 461-473.

(8) Dabrowska, J.; Brylinski, M., Stereoselectivity of 8-OH-DPAT toward the serotonin 5-HT1A receptor: biochemical and molecular modeling study. *Biochem Pharmacol* **2006,** *72*, 498-511.

(9) Huang, S.; Xu, P.; Shen, D. D.; Simon, I. A.; Mao, C.; Tan, Y.; Zhang, H.; Harpsoe, K.; Li, H.; Zhang, Y.; You, C.; Yu, X.; Jiang, Y.; Zhang, Y.; Gloriam, D. E.; Xu, H. E., GPCRs steer G(i) and G(s) selectivity via TM5-TM6 switches as revealed by structures of serotonin receptors. *Mol Cell* **2022,** *82*, 2681-2695 e6.

(10) Franchini, S.; Prandi, A.; Baraldi, A.; Sorbi, C.; Tait, A.; Buccioni, M.; Marucci, G.; Cilia, A.; Pirona, L.; Fossa, P.; Cichero, E.; Brasili, L., 1,3-Dioxolane-based ligands incorporating a lactam or imide moiety: structure-affinity/activity relationship at alpha1-adrenoceptor subtypes and at 5-HT1A receptors. *Eur J Med Chem* **2010,** *45*, 3740-51.

(11) McKune, C. M., Watts, S. W., Characterization of the serotonin receptor mediating contraction in the mouse thoracic aorta and siganl pathway coupling. *J Pharmacol Exp Ther.* **2001,** *297*, 88-95.

(12) Klein, M. T.; Dukat, M.; Glennon, R. A.; Teitler, M., Toward selective drug development for the human 5-hydroxytryptamine 1E receptor: a comparison of 5-hydroxytryptamine 1E and 1F receptor structure-affinity relationships. *J Pharmacol Exp Ther* **2011,** *337*, 860-7.

(13) Matyus, P., Varga, I., Mezei, A., Behr, A., Simay, A., Haider, N., Boros, S., Bakonyi, A., Horvath, E., Horvath, K., Novel pyridazino[4,5-b][1,5]oxazepines and - thiazepines as 5-HT1A receptor ligands. *Bioorganic & Medicinal Chemistry Letters* **1997,** *7*, 2857-2862.

(14) Blair, J. B.; Kurrasch-Orbaugh, D.; Marona-Lewicka, D.; Cumbay, M. G.; Watts, V. J.; Barker, E. L.; Nichols, D. E., Effect of ring fluorination on the pharmacology of hallucinogenic tryptamines. *J Med Chem* **2000,** *43*, 4701-10.

(15) Castriconi, F.; Paolino, M.; Giuliani, G.; Anzini, M.; Campiani, G.; Mennuni, L.; Sabatini, C.; Lanza, M.; Caselli, G.; De Rienzo, F.; Menziani, M. C.; Sbraccia, M.; Molinari, P.; Costa, T.; Cappelli, A.,

Synthesis and structure-activity relationship studies in serotonin 5-HT4 receptor ligands based on a benzo[de][2,6]naphthridine scaffold. *Eur J Med Chem* **2014,** *82*, 36-46.
(16) Eglen, R. M.; Bley, K.; Bonhaus, D. W.; Clark, R. D.; Hegde, S. S.; Johnson, L. G.; Leung, E.; Wong, E. H., RS 23597-190: a potent and selective 5-HT4 receptor antagonist. *Br J Pharmacol* **1993,** *110*, 119-26.
(17) Langlois, M., Zhang, L., Bre'mont, B., Shen, S., Manara, L., Croci, T., Design of a potent 5-HT4 receptor agonist with nanomolar affinity. *Bioorganic & Medicinal Chemistry Letters* **1994,** *4*, 1433-1436.
(18) *RDKit: Open-source cheminformatics.* https://www.rdkit.org.
